# Effects of temporal, spatial, and environmental factors on ciliates community in northeastern South China Sea, with notes on co-occurrence patterns of environment, phytoplankton, and ciliate

**DOI:** 10.1128/spectrum.01247-24

**Published:** 2024-11-29

**Authors:** Weiwei Liu, Zijun Cheng, Shaowei Wen, Gang Li, Zhixin Ke, Daning Li, Yehui Tan

**Affiliations:** 1 Key Laboratory of Tropical Marine Bio-resources and Ecology, Guangdong Provincial Key Laboratory of Applied Marine Biology, South China Sea Institute of Oceanology, Chinese Academy of Science, Guangzhou, China; 2 University of Chinese Academy of Sciences, Beijing, China; 3 State Key Laboratory of Tropical Oceanography, South China Sea Institute of Oceanology, Chinese Academy of Sciences, Guangzhou, China; Institut Ruder Boskovic, Zagreb, Croatia

**Keywords:** deep sea, diversity, microplankton, microbial food web, network, protozoa

## Abstract

Little is known about temp-spatial variations and determining mechanism of microplankton community from continental shelf to deep basin. Here, the distribution and determinants of ciliates in the northeastern South China Sea were investigated in summer and winter. We found that the alpha diversity were generally similar, but community composition showed clear difference in summer and winter. For spatial dimension, the alpha diversity significantly decreased from neritic water to deep, and the variations of community composition were only detected between upper and deep water. The determinants of community varied depending on the research scale. For entire community, the variations were higher explained by environmental factors than by temp-spatial factors. Specially, physical factors were the main determinants, which may attribute to the effects of physical processes occurred in the research area. For the three spatial subareas, season showed more significant influences on communities than other factors. Network analyses revealed that the ciliate exhibited more relationships with phytoplankton than with environment factors. Diatom and physical factors accounted for most of the ciliate-phytoplankton-environment co-occurrence relationships, suggesting their significant influences to the community stability of the microbial food web. This study improves our understanding for the mechanisms of community structuring of microzooplankton in the open sea across both space and time.

**IMPORTANCE:**

Ciliates are important components of microplanktons and play a crucial role in global nutrient cycling. There is still large knowledge gap on their structuring mechanisms from continental shelf to deep basin. We revealed the distribution patterns of ciliate community in neSCS and assessed the relative contribution of temporal, spatial, and environmental factors for their community structuring. Compared with temp-spatial factors, environmental factors take more responsibilities for the ciliate distribution. Among the environmental factors, physical factors showed the largest contribution to community variation. Moreover, the ciliate-environment co-occurrence pattern showed that physical factors contributed more for their relationships. These findings suggested that the physical processes play key roles in temp-spatial dynamics of ciliate community in open ocean.

## INTRODUCTION

Microplanktons are the foundation of marine production and have substantial impacts on biogeochemical cycles (1). As the important components of microplanktons, ciliates usually dominate the microzooplankton community and inhabit a great variety of marine environments (2, 3). Feeding on bacteria and nanoflagellates, and serving as the food of copepods and other small metazoans, ciliates play a crucial role in microbial food webs as the link between primary producers and higher trophic levels (4, 5).

The spatial and temporal distribution characteristics of microplanktons have always been the focus of marine ecology research. A growing number of studies have recently indicated that the distribution rules of ciliates depend on study scale and ecosystem types (6–10). For example, in the intertidal area of South China coast, the ciliate communities separated based on habitat groups, while in the offshore area, they clustered by geographic groups (11). A study on biogeographical patterns of benthic ciliates in coasts of China showed that the community are driven by local environments (12). However, the Tara Oceans Voyage investigation found that most ciliates are global distributed and local species only represented 27% of the total richness (2).

Revealing the determinants of microbial communities is of significance to understand the processes and mechanisms that generate and maintain microbial diversity (13, 14). The ciliate community reportedly exhibits a strong relationship with the environmental factors. Physical factor (e.g., salinity, temperature, and dissolved oxygen) were identified as significant factors impacting the ciliate community. For example, the growth and reproduction of ciliates are strongly correlated with temperature (15). Dissolved oxygen was known as a dispersal barrier for ciliates and led to allopatric speciation in isolated habitats (16). Chemical factors such as nitrate and phosphate were the main nutrient sources, which were also revealed to have a strong relationship with ciliates (17). The reasons are twofold: nutrients can enhance phytoplankton growth and, thus, indirectly influence heterotrophic ciliates that feed on phytoplankton, and nutrients can be directly taken up by autotrophic ciliates (18, 19). Moreover, biotic factors, such as phytoplankton and bacteria, can effect the ciliate communities by bottom-up control as the food resource of ciliates (20). In addition to environmental deterministic, stochastic processes induced by temporal and spatial variations also play important roles in driving ciliate communities. For example, dispersal limitation caused by spatial distance was considered the main reason for ciliate community variations in Taiwan Strait (21). The temporal variation can impact the efficiency of immigration and cell division, leading to seasonal turnover of ciliate community in Ganges River Estuary (22). The investigations on the biogeography patterns of ciliates have been conducted in different areas of the world (2, 23–25). However, few studies focus on their high-resolution distributions in open ocean in large scale (e.g., hundreds of kilometers long), and the information on the main driver of their community assembly is hardly available.

The South China Sea (SCS) is the largest marginal sea of the western Pacific Ocean. With a maximum depth of over 5,000 m, the northeastern South China Sea (neSCS) had a broad continental shelf shallower than 200 m linked to the deep basin by a steep shelf slope (26, 27). Impacted by monsoon systems, the circulation patterns displayed temp-spatial variations in the neSCS (28). These water masses bring the variations of physical and chemical conditions and further present a significant influence to the distribution of microplanktons. The temp-spatial variation of other microplanktons has been commonly revealed in the SCS. For example, the ichthyoplankton assemblages were identified into offshore and inshore groupings in the neSCS (29). The dominant grouping of the phytoplankton community was significantly different in summer and winter (30). However, less is known about the temp-spatial patterns of ciliate communities.

We speculated that, with the strong environmental dynamics induced by complex physical process, the distribution of ciliates will present significant temp-spatial patterns in the neSCS like other microplankton did. Moreover, the relative importance of various determinative factors for ciliate communities may change depending on season and area. Here, for the first time, we provide comprehensive information on the temp-spatial distribution of ciliate communities in the neSCS at fine spatial scale by sampling with full coverage of this area. By comparing the contributions of environmental or temp-spatial factors to community assembly, this study reveals the determining mechanism of ciliate distribution in the neSCS.

## MATERIALS AND METHODS

### Study sites and Sampling

**Fig 1:**
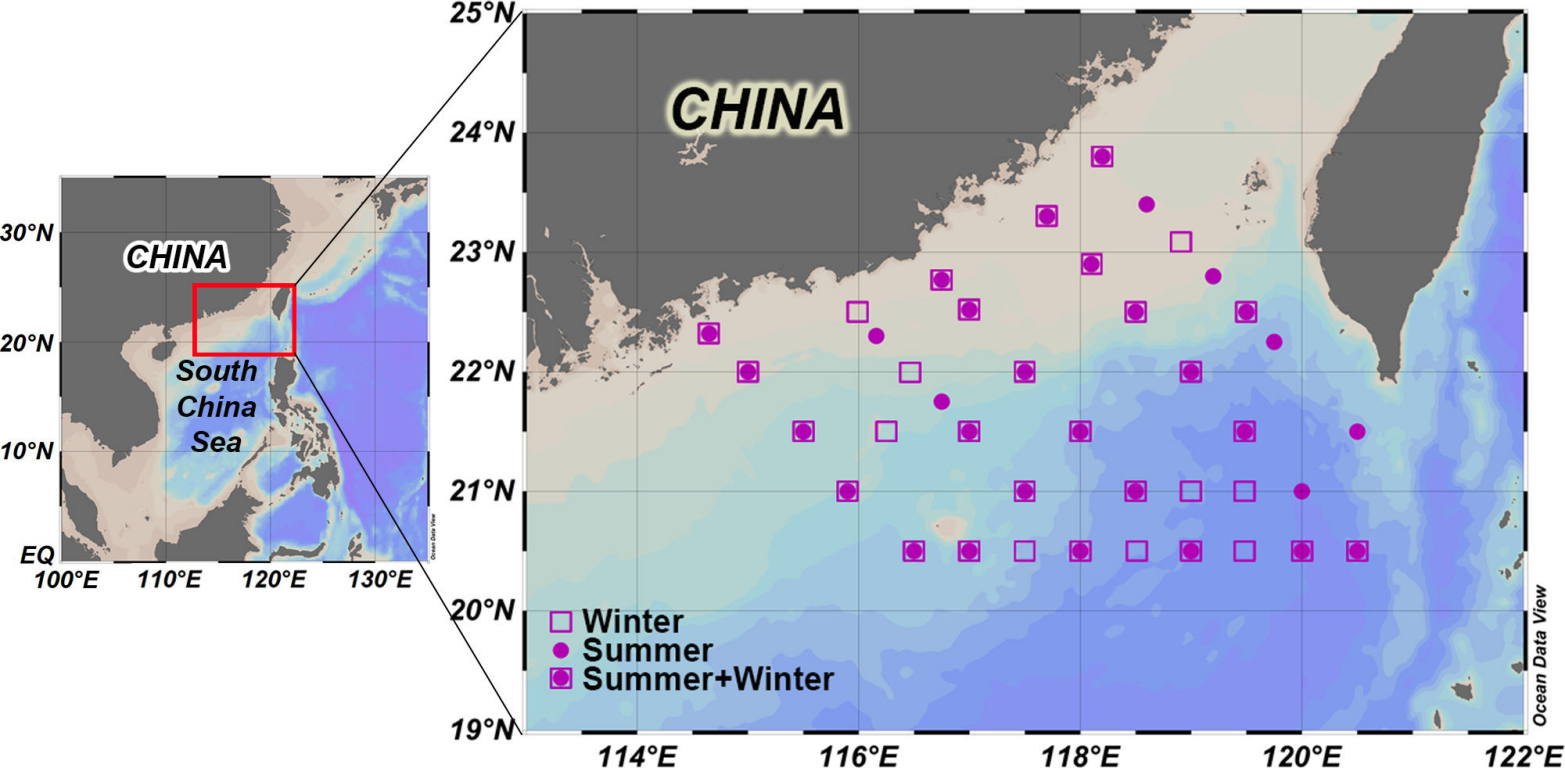
The sampling stations in the neSCS of summer 2015 (round) and winter 2016 (square). The maps were generated with the program Ocean Data View.

Two cruises were conducted to document ecological and environmental parameters by research vessel R/V Shiyan 3 in the neSCS (113–122°E, 18–24.5°N). This study investigated a total of 31 sampling stations (169 samples) during the summer cruise (3–19 August 2015) and 33 sampling stations (173 samples) during the winter cruise (28 February to 20 March 2016; Fig. 1). Water samples were collected from the surface down to the mesoplelagic zone (0, 25, 50, 75, 100, 200, 300, 500, 1,000, and 1,500) using eight L-Niskin bottles mounted on a CTD rosette (Sea-Bird 911 plus). To determine the ciliate community, the water samples (2 L) were fixed immediately with acid Lugol’s iodine solution (2% final concentration) and stored in cold and dark conditions until analysis.

### Environmental Parameters

Standard methods for environmental variables including salinity (Sal), water temperature (Tem), pH, dissolved oxygen (DO), inorganic nitrogen (Nox: nitrate plus nitrite), soluble reactive phosphorus (SRP), active silicon (Si), abundance of picoplanktons Synechococcus (Syn), Picoeukaryotes (Peuk), and Prochlorococcus (Pro), as well as size fractioned chlorophyll a concentration, i.e., Microplankton Chla (MChla >20 µm); Nanoplankton Chla (NChla, 3–20 μm); Picoplankton Chla (PChla, <3 µm) have been detailed in previous publications (31).

### Ciliate identification and enumeration

For identification and enumeration of ciliates, the Lugol’s fixed sample was concentrated by settling to 50 mL. Subsamples (1, 5, 10, or 50 mL, depending on the cell density) were taken into a counting chamber, and all ciliates in the chamber were identified and counted under an inverted microscope at magnifications of 200×. Following this procedure, at least 10 individuals of the dominant taxa and fewer of rare taxa were enumerated in each subsample. Tintinnids were identified by lorica and cell morphology according to reference (32). Aloricate ciliates were identified following (33, 34). All ciliates were identified to species level. Uncertain individuals (about 2% of all observed individuals) were picked out and identified using protargol impregnation after re-fixation with Bouin’s solution (35). Classification followed (3). Species composition and abundance in each sample were recorded for each season, forming the community structure matrices that were then used for community analyses. Moreover, we clarified the ecological trait composition of ciliates in terms of feeding habits and trophic types. The species were assigned to five feeding habits, which comprised detritivores, bacterivores, algivores, raptors, and non-selectives according to the original sources in which the species were described, as well as the broader literature (3, 36, 37). For trophic composition, the designation of species as being hetreotrohic or mixotrophic was made according to the literature (3, 23, 34, 38)

### Statistical analyses

The temp-spatial distribution of ciliates communities were analyzed based on two temporal groupings, i.e., summer and winter, and three spatial groupings, i.e., neritic water (bottom depth of sample site was less than 200 m), upper oceanic water (bottom depth of sample site was more than 200 m and water depth of sample was less than 200 m), deep oceanic water (bottom depth of sample site was more than 200 m and water depth of sample was more than 200 m). Tukey’s HSD test was performed to test the differences in ciliate abundances and alpha diversity indexes including species richness and Shannon among groupings. Non-metric multidimensional scaling ordination (NMDS) analyses were conducted and analysis of similarity (ANOSIM) was used to statistically test for significant differences of ciliate communities among groupings based on Bray-Curtis dissimilarity. Spearman’s rank coefficients were calculated to relate ciliate abundance/alpha diversity indexes and all environmental factors. Mantel tests were used to explore the relationships between ciliate communities and environmental variables. Variation partitioning analysis (VPA) was used to evaluate the relative contribution of the environmental and temp-spatial variables in shaping ciliate communities with adjusted *R*2 coefficients based on redundancy analysis (RDA) or canonical correspondence analysis (CCA). Temp-spatial variables are composed of season, vertical (Depth) and horizontal (a set of variables was generated using PCNM analysis based on the longitude and latitude of sampling sites) variable, and environmental variables are composed of food resource factors (Food: referring to MChla, PChla, NChla, Syn, Peuk, and Pro), physical factors (Phy: referring to Sal, Tem, pH, DO), and chemical factors (Chem: referring to Nox, SRP, Si). Before the RDA or CCA analysis, a forward selection was conducted to select significant explanatory variables (*P* < 0.05) for further analyses. All analyses were performed using the “vegan” package in R (39).

Two co-occurrence networks were constructed. One was constructed based on all data set of ciliates community to understand the relationships among ciliates. Another was based on the combined data set of environmental factors, ciliates, and phytoplankton communities to assess the relationships among the three components. The phytoplankton data were obtained from the previous study (30). To reduce noise and complexity of the data sets, ciliate species present in more than one samples with relative abundance more than 0.5% were retained for the construction of networks. The pairwise Spearman’s rank correlations (*r*) among the data were calculated by “psych” package in R, and only correlations with |*r*| > 0.2 and *P* < 0.05 were included in the network. Network visualization, modular analysis, and node level topological properties (i.e., degree) were completed using Gephi version 0.9.2. In addition, 1,000 Erdös–Réyni random networks were obtained, which had the same number of nodes and edges as the real networks, with each edge appearing with the same probability of being assigned to any node (40). Topological characteristics of both real and random networks were calculated and compared, including clustering coefficient, modularity, and average path length. Small-word coefficient (*r*) was calculated as mentioned in a previous study (41). To help explain the module characters, the niche breadth of each module was estimated using Levins’ niche breadth index (42).

## RESULT

### Environmental characterization

Principal component analysis (PCA) of environmental parameters revealed that the samples in the two seasons could not be distinguished, but these in the three spatial groupings were clearly separated (Fig. S1), which suggest that the variations of environmental parameters were not significant in seasonal dimensions but significant in spatial dimensions. In addition, variations occurring horizontally were related mainly to increases in most Chla parameter and a decrease in salinity from neritic water to upper oceanic water area, while vertical variations down the water column were linked to increases in all the chemical parameters and decreases in most physical parameters like DO, temperature, and pH from upper to deep water.

### Distribution of alpha diversity and abundance

Altogether 263 ciliates species, assigned to 10 classes/subclasses, 20 orders, 48 families, and 90 genera, were detected. The ciliate communities were dominated by the subclasses Oligotrichia (relative abundance 36.3%) and Choreotrichia (34.5%), followed by classes Prostomatea (17.1%), Litostomatea (9.4%), and Oligohymenophorea (1.5%), whereas the relative abundance of other taxa (e.g., Euplotia, Hypotrichia, Phyllopharyngea, Protocruziea, Percolatea) were each less than 1%. At family rank, Strombidiidae and Strobilidiidae (31.1% and 21.9%, respectively) kept a high proportion in the community.

**Fig 2:**
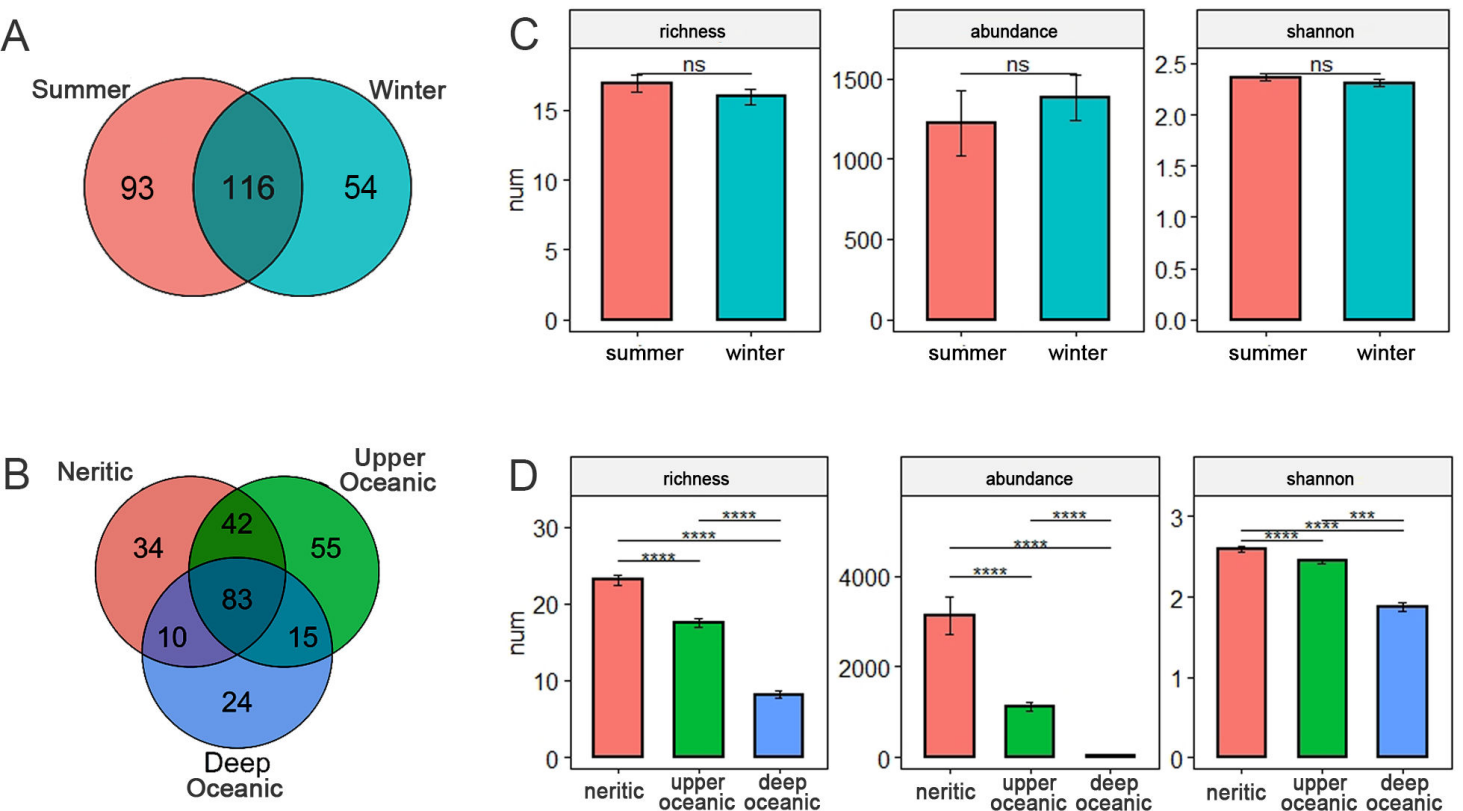
Temp-spatial variations of ciliates diversity and abundance. (**A, B**) Venn diagram showing the numbers of unique and shared species in summer and winter (**A**) and in three spatial zones (**B**). (**C, D**) Comparison of ciliate species richness, abundances, and shannon in two seasons (**C**) and in three spatial zones (**D**). Pairwise comparisons are tested using Tukey’s HSD test. ****P < 0.001, ***P < 0.01, and ns, not significant.

The number of species found in summer was higher than winter (i.e., 209 vs 170 species, among which 116 species were shared by both seasons Fig. 2A). But the average species richness of samples were similar in the two seasons (Fig. 2C). And the average shannon and abundance did not show seasonal variation.

Comparison among spatial groupings showed that the number of species was highest in upper oceanic water and lowest in deep water (i.e., 195 in upper oceanic water vs 169 in neritic water vs 132 in deep oceanic water, Fig. 2B), and the number of shared species between neritic water and upper oceanic water was much higher than that between deep and upper oceanic water (125 vs 98), suggesting that the variations of community composition were more significant in vertical than horizontal directions. In terms of the average alpha diversity of samples, all the species richness, shannon, and abundance significantly decreased from neritic to deep water (Fig. 2D).

### Distribution of beta diversity

**Fig 3:**
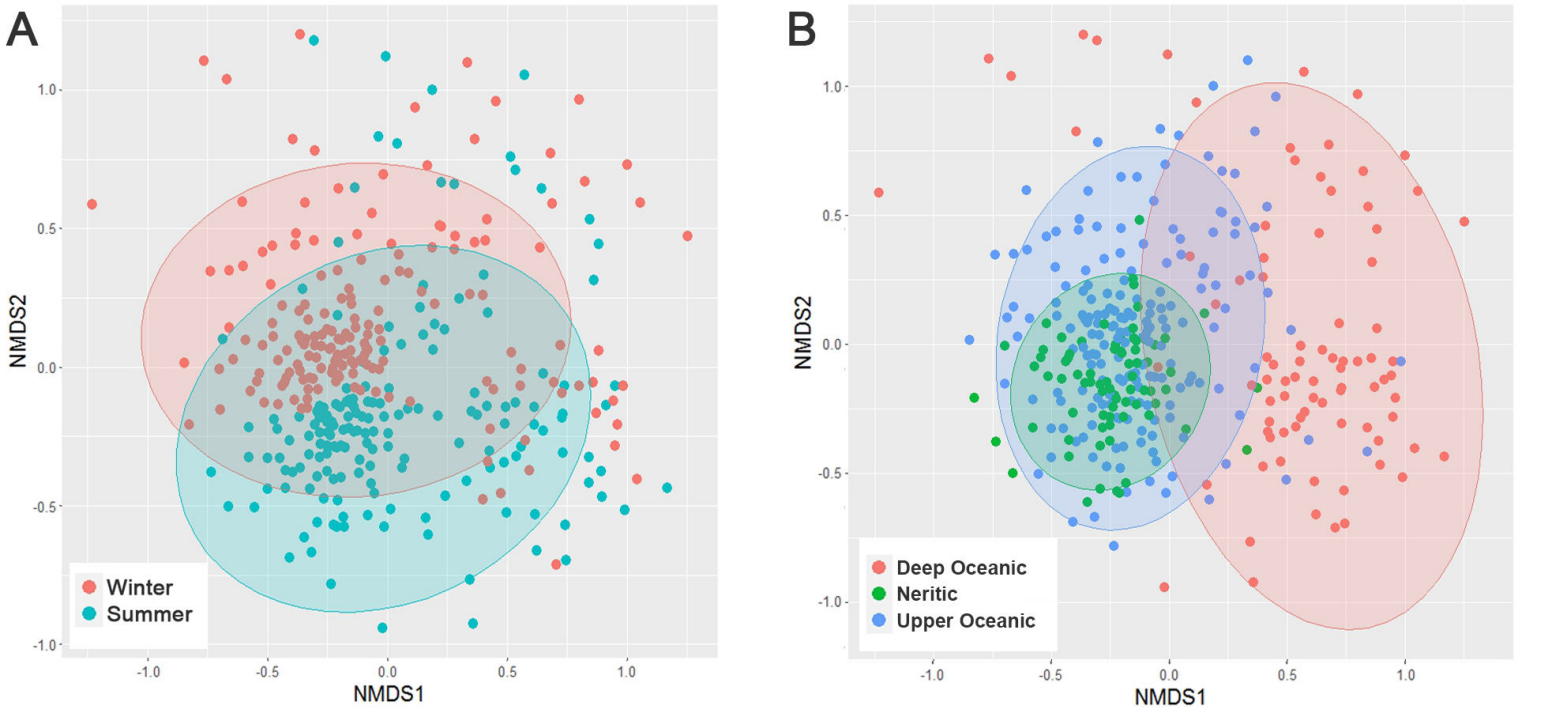
Non-metric multidimensional scaling (NMDS) ordination of communities based on Bray-Curtis dissimilarity, samples coded by color in the two seasons (**A**) and three spatial zones (**B**). Stress value: 0.2677.

The NMDS showed that the ciliates communities can be separated based on seasonal and vertical groupings, but the horizontal variation between neritic and upper oceanic water cannot be clearly observed (Fig. 3). However, ANOSIM indicated that all the seasonal and spatial groupings can be identified (Table 1). The community dissimilarity between upper oceanic water and deep water was highest, followed by that between winter and summer, and that between neritic water and upper oceanic water was the lowest (Fig. S2), indicating that the variations of community in vertical grouping were more significant than seasonal and horizontal groupings. In addition, the seasonal variations were also found in each spatial zone (Table 1), indicating that the seasonal turnover of community happened locally.

**TABLE 1:** Analysis of similarities (ANOSIM) of ciliate communities among season and spatial groupings

Pairwise tests		R	P
Summer vs winter		0.065^*a*^	<0.001
Upper oceanic water vs deep oceanic water		0.743^*a*^	<0.001
Neritic water vs upper oceanic water		0.076^*b*^	0.006
Neritic water	Summer vs winter	0.194^*a*^	<0.001
Upper oceanic water	Summer vs winter	0.124^*a*^	<0.001
Deep oceanic water	Summer vs winter	0.127^*a*^	<0.001

^*a*^ *P* < 0.001.

^*b*^ *P* < 0.01.

**Fig 4:**
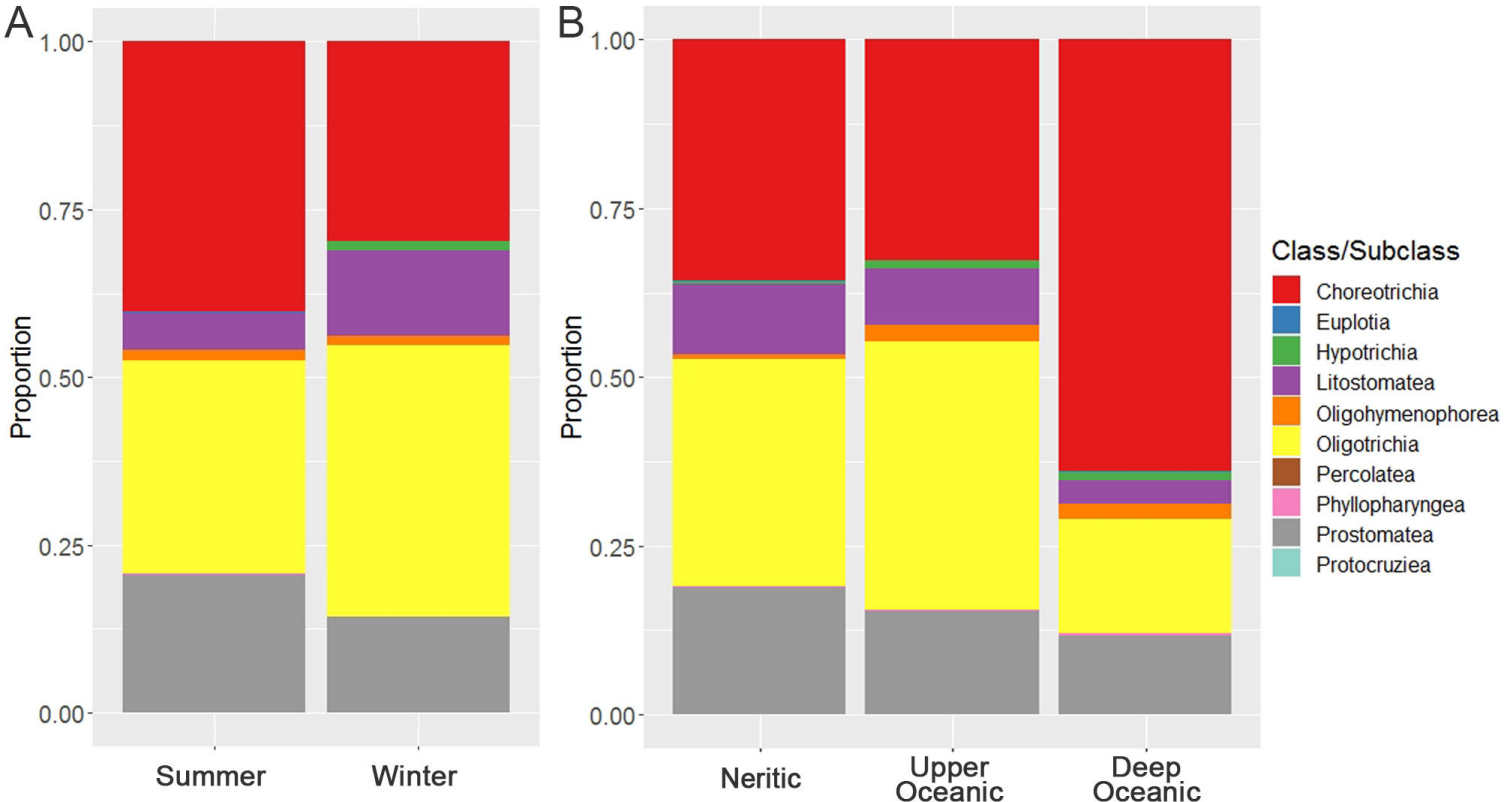
Taxonomic composition at class/subclass level of ciliates in two seasons (**A**) and in three spatial zones (**B**).

The taxonomic composition displayed differences between the two seasons (Fig. 4A). For example, the proportions of Choreotrichia (67.7% vs 60.2%) and Prostomatea (13.2% vs 10.4%) significantly higher in summer than winter, whereas those of Litostomatea (3.1% vs 3.9%) and Oligotrichia (13.5% vs 20.1%) were lower in summer (Fig. 4A). For spatial groupings, the taxonomic composition showed significant variation between deep water and neritic or upper oceanic water, whereas that in neritic water and upper oceanic water were generally similar (Fig. 4B). For example, the proportions of Choreotrichia (64.0% vs 35.7% vs 32.7%) were higher in deep than neritic and upper oceanic water, while those of Prostomatea (11.8% vs 18.8% vs 15.2%), Litostomatea (3.5% vs 10.4% vs 8.3%), and Oligotrichia (16.8% vs 33.6% vs 39.9%) were lower in deep than neritic and upper oceanic water. The significant variations of community in vertical groupings can also be observed in feeding and trophic type composition (Fig. S3). From neritic and upper oceanic water to deep water, the proportion of algafeeding ciliates significantly decreased but that of detric feeding type increased, whereas the proportion of mixotrophic ciliates significantly decreased.

### Correlations between diversity and environmental factors

**Fig 5:**
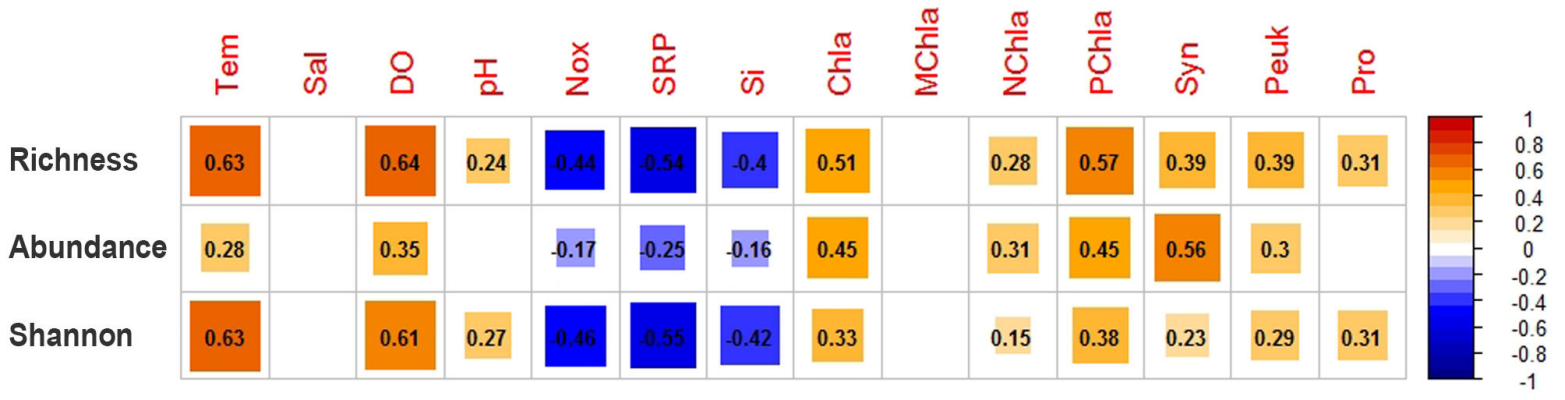
Correlation analyses between environmental variables and ciliate species richness, abundance, and shannon. The number and color gradient represent Pearson’s correlation coefficients. The blank representing correlation is not significant (*P* > 0.05). Tem, temperature; Sal, salinity; DO, dissolved oxygen; Nox, inorganic nitrogen; SRP, soluble reactive phosphorus; Si, active silicon; Chla, total chlorophyll a; MChla, microplankton chlorophyll a (>20 µm); NChla, nanoplankton chlorophyll a (3–20 μm); PChla, picoplankton chlorophyll a (<3 µm); Syn, Synechococcus; Peuk, Picoeukaryotes; and Pro, Prochlorococcus.

Spearman’s correlation analyses showed that the influences of environmental factors on species richness, shannon, and abundance of entire community were similar, i.e., they were usually negatively correlated with chemical factors such as Nox, SRP, Si, and positively correlated with physical (i.e., Tem, DO) and food factors (i.e., Chla, NChla, PChla, Syn, Peuk) (Fig. 5). The effects of environmental factors on alpha diversity of each seasonal and spatial group were also analyzed, respectively (Fig. S4). Both summer and winter subcommunities displayed similar results with the entire community. For the three subareas, the correlation results were significantly different in terms of the numbers of environmental factors with significant effect. Compared with neritic water, more chemical and food factors with significant correlations were found in upper oceanic water, indicating that the effects of chemical and food factors on alpha diversity enhanced from neritic to oceanic water. In deep water, most food factors did not show significant correlations with species richness, abundance, and shannon, indicating that the influence of food on alpha diversity was little there.

The mantel test showed that the dissimilarity of the entire community was significantly correlated with all physical and chemical factors, as well as some food factors such as Chla, PChla, and Peuk (Table 2). Most factors correlated with summer subcommunity, whereas most food factors didn’t correlated with winter subcommunity. For spatial groupings, the factors with significant effects on the community were few especially in deep water. From neritic to upper oceanic water, the numbers of chemical factors with significant correlations to the community increased. No food factors showed significant effects on subcommunity in deep water.

**TABLE 2:** Mantel tests for the correlation between community similarity and environmental variables using Pearson’s coefficient^*a*^

	Entire			Summer		Winter		Neritic water		Upper oceanic water		Deep oceanic water	
	R	P	R		P	R	P	R	P	R	P	R	P
Tem	**0.527**	**<0.001**	**0.632**		**<0.001**	**0.536**	**<0.001**	**0.291**	**<0.001**	**0.223**	**<0.001**	**0.105**	**0.034**
Sal	**0.061**	**0.038**	**0.103**		**0.005**	0.068	0.081	**0.272**	**<0.001**	−0.010	0.591	0.078	0.167
DO	**0.511**	**<0.001**	**0.573**		**<0.001**	**0.615**	**<0.001**	**0.212**	**<0.001**	**0.236**	**<0.001**	**0.198**	**0.001**
pH	**0.166**	**<0.001**	**0.174**		**<0.001**	**0.195**	**<0.001**	**0.148**	**0.046**	**0.088**	**0.021**	0.076	0.175
Nox	**0.354**	**<0.001**	**0.430**		**<0.001**	**0.281**	**<0.001**	0.055	0.224	**0.111**	**0.016**	0.012	0.419
SRP	**0.442**	**<0.001**	**0.499**		**<0.001**	**0.409**	**<0.001**	**0.157**	**0.012**	**0.226**	**<0.001**	**0.127**	**0.013**
Si	**0.294**	**<0.001**	**0.347**		**<0.001**	**0.330**	**<0.001**	−0.013	0.520	**0.097**	**0.030**	**0.195**	**0.003**
Chla	**0.150**	**<0.001**	**0.164**		**<0.001**	**0.260**	**<0.001**	0.120	0.096	**0.112**	**0.003**	−0.041	0.682
MChla	−0.016	0.571	0.038		0.205	−0.028	0.611	0.014	0.363	−0.063	0.938	NA	NA
NChla	0.033	0.165	**0.087**		**0.029**	−0.019	0.623	0.080	0.176	−0.030	0.769	−0.045	0.713
PChla	**0.090**	**0.001**	**0.247**		**<0.001**	−0.012	0.604	0.090	0.091	0.007	0.403	−0.062	0.783
Syn	0.045	0.069	**0.140**		**0.001**	−0.047	0.872	**0.168**	**0.013**	0.076	0.051	−0.105	0.949
Peuk	**0.068**	**0.014**	**0.114**		**0.002**	0.067	0.059	0.014	0.393	**0.130**	**0.005**	−0.035	0.671
Pro	−0.041	0.940	−0.020		0.688	−0.024	0.723	−0.063	0.798	−0.043	0.857	−0.092	0.911

^*a*^ Bold values indicate statistical significance (P<0.05).

**Fig 6:**
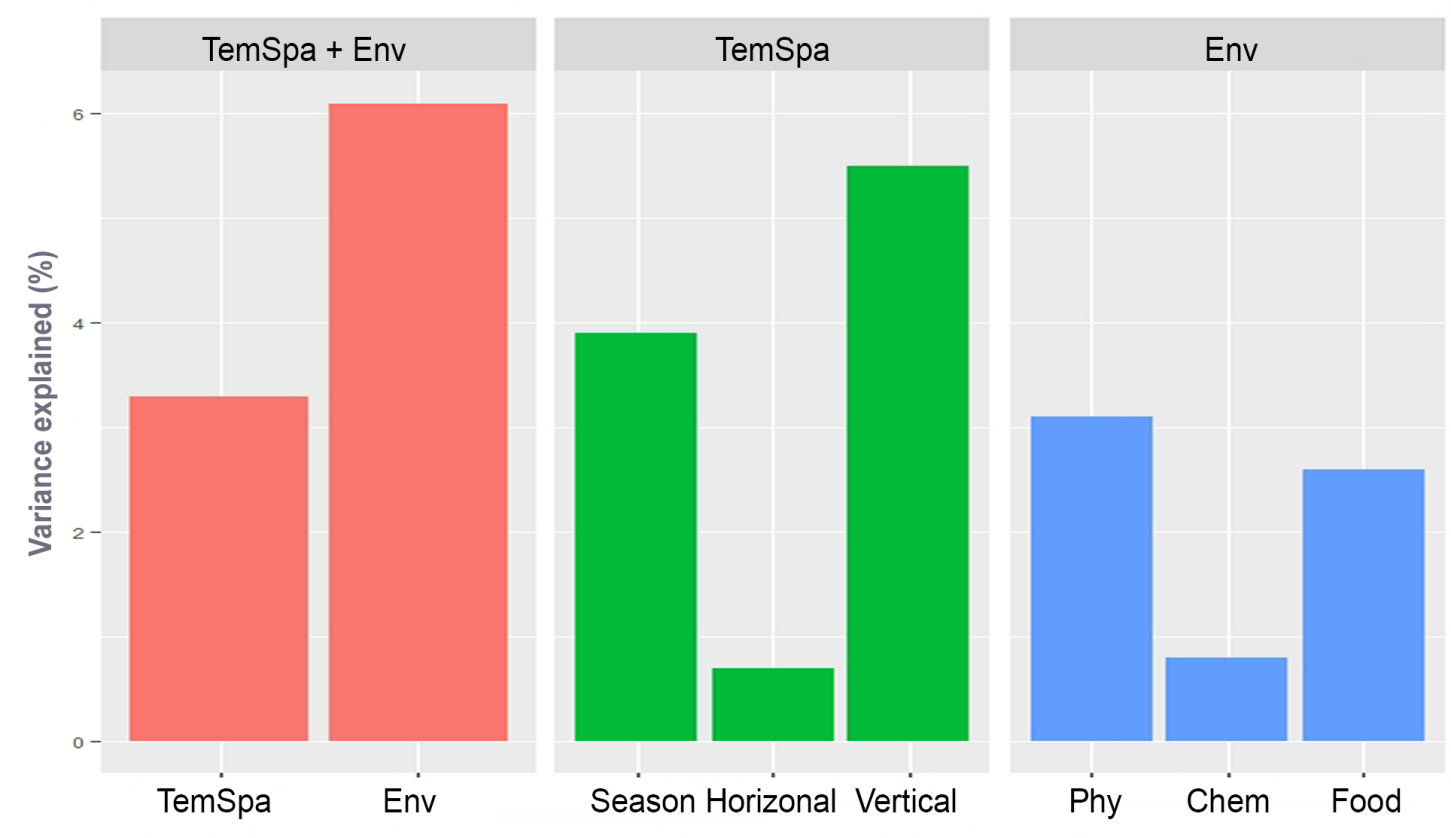
The explanatory power of various drivers for ciliate community revealed by variation partitioning analysis. (**A**) Comparisons of the variations explained by temp-spatial (TemSpa) and environmental (Env) factors correspond to the community; (**B**) comparisons of the variations explained by season, horizontal and vertical spatial factors; (**C**) Comparisons of the variations explained by physical (Phy), chemical (Chem), and food factors. TemSpa: temp-spatial factors referring to season, depth (vertical) and PCNM (horizontal); Env, environmental factors referring to physical (Sal, Tem, pH, DO), chemical (Nox, SRP, Si), and food (MChla, PChla, NChla, Syn, Peuk, and Pro) variables. Forward selection procedures were used to select the best subset of variables explaining community variation, respectively.

In addition, the relative contributions of the environmental and temp-spatial variables in the community distribution were compared by VPA (Fig. 6). For the entire community, the contribution of environmental factors to the community was higher than that of temp-spatial factors (with an explanation 6.1% vs 3.3%). Within temp-spatial factors, the vertical factors explained higher variation of community than season did (5.5% vs 3.9%), and horizontal factors explained the lowest (0.7%). Within environmental factors, physical factors showed largest contribution to the community variation (3.1%), followed by food (2.6%), while chemical factors had smallest contribution (0.8%). For the spatial subcommunities, the results in each local area showed some differences from the entire community in terms of main contributors (Fig. S5). For example, the most important effectors in TemSpa factors were vertical factors for the entire community but became season for the three spatial subcommunites, and the environmental factors showed higher contributions than TemSpa factors for the entire community, but the result was reversed in upper oceanic water and deep water. In addition, notably, no horizontal, physical, and food factors were significantly related to the distribution of subcommunity in deep water according to CCA result and, thus, did not give explanation to it.

### Co-occurrence relationships of ciliates, phytoplanktons, and environmental factors

**Fig 7:**
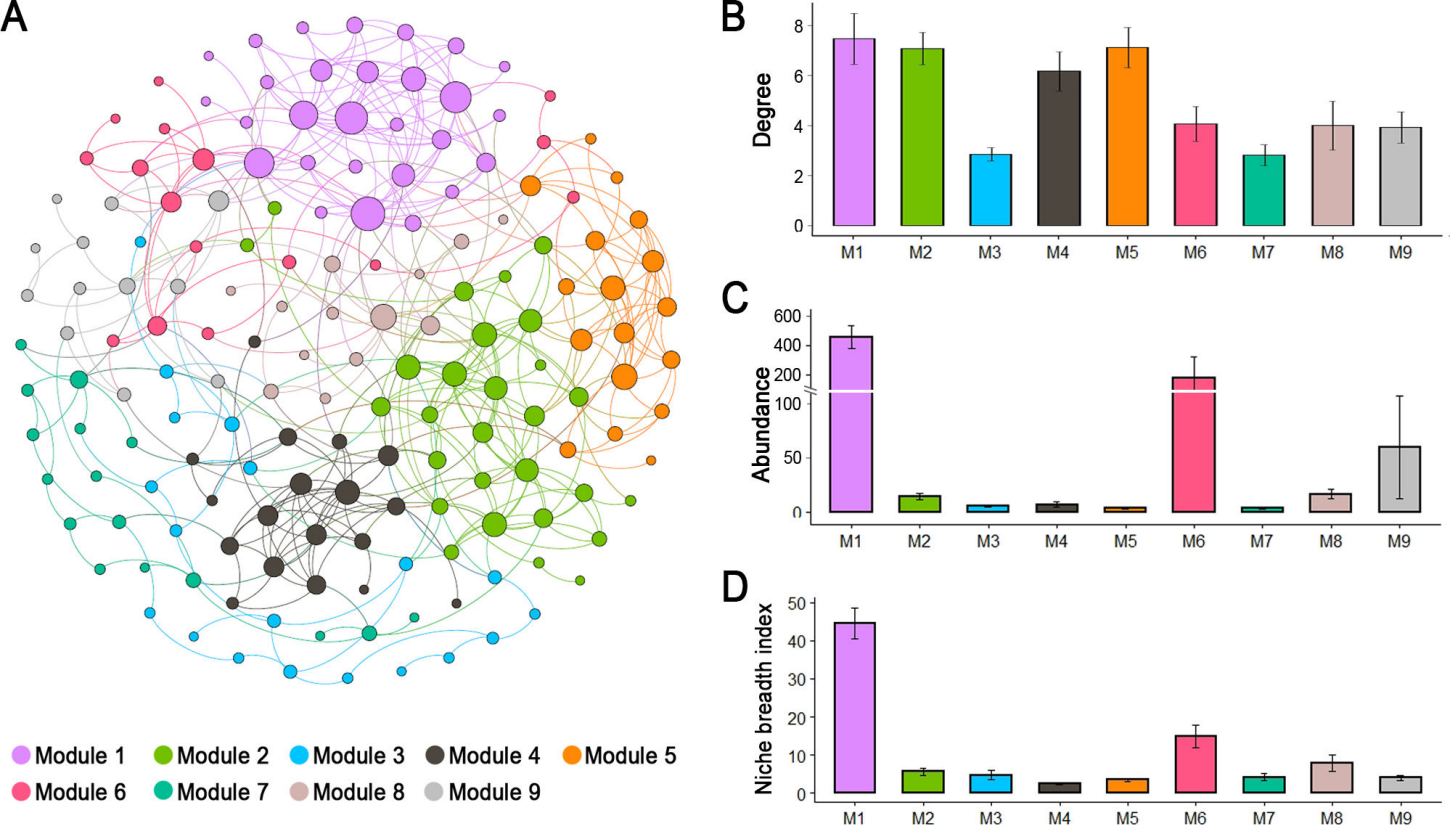
The co-occurrence patterns among ciliate species revealed by network analysis. (**A**) Network revealing the modular associations. The size of each species (node) is proportional to the number of connections (i.e., degree). (**B**) Comparison of average degree of nodes among the different modules. (**C**) Comparison of average abundance of nodes among the different modules. (**D**) Comparison of average niche breath index of nodes among the different modules.

The network of ciliate community consisted of 167 nodes (species) and 451 edges (correlations) (Fig. 7). The observed modularity, average clustering coefficient, and average path length were much higher compared to their corresponding values from Erdös–Réyni random networks, and the *r* values of networks were all higher than 1 (Table S1), implying the network exhibited a “small-world” property. The network was parsed into 9 major modules, of which modules 1 and 2 contained more nodes (each accounted for 16.77% of the whole network nodes) than others. We analyze the temporal and spatial preference of the species in each module based on abundance (Fig. S6). The results showed that most modules were specific (relatively more abundant) to neritic and upper oceanic water, but for season, some modules (M2, M4, M5) had higher abundance in summer, while some (M9) showed the reverse. In terms of network property, module 1 had higher value of degree than other modules (Fig. 7B). Furthermore, the higher abundances and niches breadth were found for module 1 (Fig. 7C and D). In addition, the taxonomic composition displayed significant variations among the modules, for example, the dominant group was Choreotrichs in M2-8 but change to Oligotrichs in M1 and Litostomats in M9 (Fig. S7).

**Fig 8:**
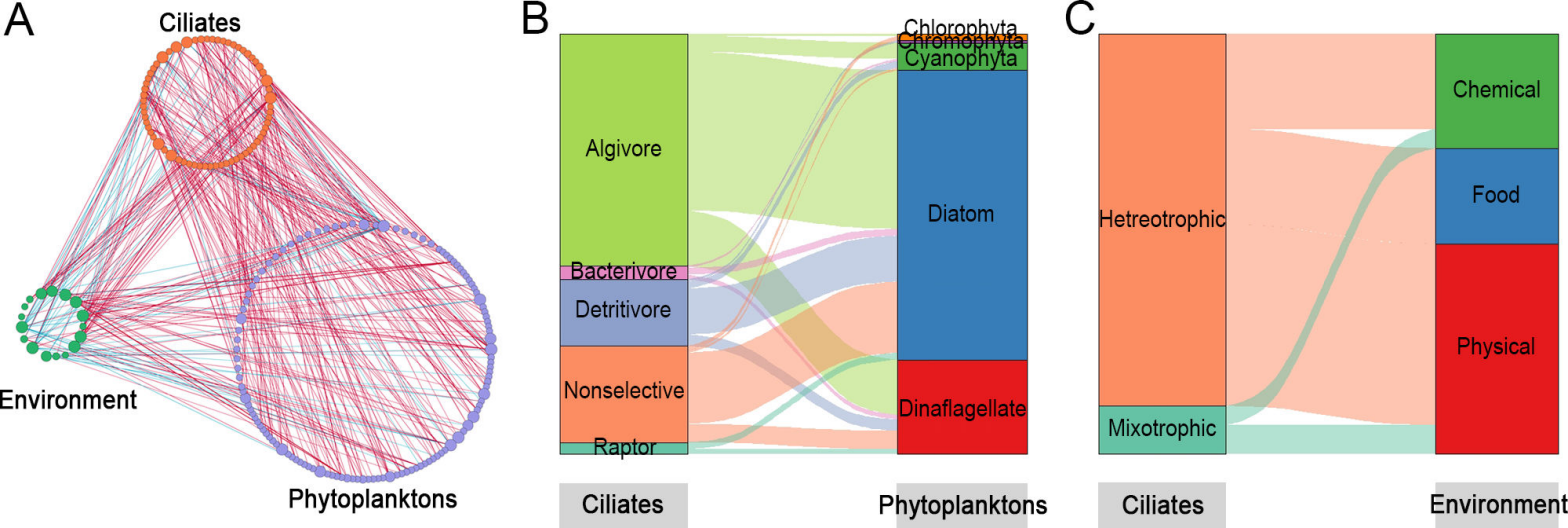
Network analysis revealing the associations among ciliates, phytoplanktons, and environmental factors. (**A**) The co-occurrence patterns among ciliates, phytoplanktons, and environmental factors, the red and blue connections stand for positive and negative correlations, respectively. (**B**) The relations between feeding habit compositions of ciliates and taxonomy compositions of phytoplanktons at the phylum level. (**C**) The relations between trophic type compositions of ciliates and groupings of environmental factors.

The integrated network of ciliate, phytoplankton, and environmental factors was also constructed. The network was consisted of 199 nodes which contain 64 ciliates, 118 phytoplankton, and 17 environmental factors (Fig. 8). Statistical analysis showed that the phytoplankton and environment variables associated with 85.9%, and 37.5% of the ciliate nodes, respectively. There were totally 686 edges in the network. And 334 edges were referring to ciliates, of which the relations between ciliates and phytoplankton were most (183), followed by ciliate internal relations (73), and ciliate-environment relations (68). Among the ciliate internal relations, the percentage of positive edges was extremely higher than the negative ones (93.3% vs 6.7% in integrated network in Fig. 8; 95.12% vs 4.88% in ciliate network in Fig. 7), suggesting that facilitation rather than competition was more common within the ciliates communities. For the relations between ciliates and phytoplankton, the most links come from algivore ciliates (feeding on alga), whereas on the phytoplankton side, diatom showed a significantly higher proportion among the relations (Fig. 8B). For the relations between ciliates and environments, the physical factors exhibited a higher contribution compared to chemical and food factors (Fig. 8C). Moreover, the relations referring food all come from hetreotrophic ciliates, and mixotrophic ciliates were only related to chemical and physical factors.

## DISCUSSION

### Temp-spatial patterns of alpha diversity and abundance attribute to the distribution of food sources

The seasonal variations of ciliate alpha diversity were commonly found in coastal area such as estuary and bays, which were thought to be resulted from the seasonal fluctuations of nutrition input from land (22, 43). In offshore water, the seasonal dynamics were rarely known. In neSCS, the typical offshore area, however, no seasonal variation of alpha diversity was detected, since species richness, abundance, and shannon did not show a significant difference between summer and winter in our results. It is well known that the species diversity and abundance of ciliate mostly depend on their food source (44), which was also confirmed by the positive correlation between alpha diversity and most food factors in our study. Because the offshore water is oligotrophic all the year, no seasonal fluctuations of nutrition occurred there (Fig. S1) (26). The concentrations of food sources are temporally stable which, thus, lead to a similar diversity and abundance in different seasons.

In contrast to the insignificant seasonal variation, spatial variation of alpha diversity among the three zones was clear. The species richness, shannon, and abundance all significantly decreased from neritic water to deep water. The same variation trend was also found in other areas such as northwest of SCS, southwestern Atlantic, and New England shelf (18, 44, 45). The nearshore area is usually under the influence of upwelling and coastal currents, which bring high nutrients and significantly enhance the standing stock of ciliate (18). The studies on ciliate distribution in the water column, especially including the mesopelagic zone, are limited (46, 47). Because the food sources such as phytoplankton and bacteria were scare below the euphotic zone, it is not surprise to find the significant lower diversity and abundance in deep than neritic water and upper oceanic water in neSCS.

### Temp-spatial variations of beta diversity related to effects of physical processes

For beta diversity, the ciliate communities from summer and winter clearly separated in our NMDS and ANOSIM results. The seasonal variations of microeukaryotic community have commonly been reported in the north Pacific Ocean and northwest Mediterranean Sea (48, 49). The seasonal fluctuations of physical environment process were considered to be responsible for the variation (50). In neSCS, the main physical processes were upwelling and SCS current in summer but changed to coastal current and Kuroshio in winter (30). These water masses brought the variations of biotic and abiotic conditions and, thus, caused the turnover of community composition between the two seasons. This was supported by our results of community composition. We found that the proportions of Oligotrichs and Litostomatea were significantly higher in winter than summer. This attributed to the effects of physical processes in two aspects. First, the physical process causes the seasonal difference in phytoplankton composition, i.e., the phytoplankton community was dominated by *Trichodesmium erythraeum* (cyanobacteria) and diatoms in summer and winter, respectively (30). As typical algivore ciliate, Oligotrichs and Litostomats preferred diatoms to *Trichodesmium erythraeum* as their prey due to the size selectivity (51) and, thus, had abundant food source in winter. Second, the dominants of Oligotrichs (e.g., *Strombidium* species) and Litostomats (e.g., *Mesodinium* species) are also mixotrophic ciliates. Compared with summer, Kuroshio intrusion is strong in winter. Kuroshio water is characterized by high transparency (52), which improves the photosynthetical active radiation level and may be conducive to the growth of mixotrophic ciliates. Combined with our mantel test results that the dissimilarity of the community were significantly correlated with all physical factors, our study indicated that the physical environment process played important roles in the seasonal variation of community.

For the spatial groupings, our NMDS did not reveal the significant separation of community between neritic and upper oceanic water. This was confirmed by our results of community composition, which showed a high proportion of shared species and similar taxonomic pattern between the two zones. The high community similarity may be attributed to the high water connectivity and strong interaction of communities between neritic and upper oceanic water driven by water mass. Our result is different from the findings in some other studies which revealed a switch of ciliates community from shelf to oceanic water (24, 53). This difference might be related to sampling scale or sample size. In previous studies, the sampling sites were sparsely distributed, and the sample amount was few (e.g., <50), which may amplify the individual deviation of samples resulted from local ecological variation. In our study, the fine scale sampling with large sample size (e.g., >200) dilutes the individual deviation and, thus, reflects the global variation. In addition, due to no effect of water mass (24, 53), the water interaction between neritic and oceanic areas was fewer in their studies, which might be another reason for their more significant community variation than neSCS. In the vertical direction, significant variation of ciliate community was displayed in our NMDS, ANOSIM, and taxonomy composition between upper and deep oceanic water. The vertical variation of community in ocean water was mostly attributed to the difference of environment characters (46). In our study, the significant environmental variation between upper and deep water was revealed (Fig. S1B). The vertical environmental variations form a wide range of niches, which, thus, were occupied by different organisms (54). Notably, the proportion of Choreotrichs in community composition remarkably increased and that of Oligotrichs decreased from upper to deep water. Similar distribution patterns of these two ciliate groups were also found in open pacific ocean (55). Choreotrichs mostly possess a lorica attached their body which gain the weight and improve the ability to sink to deep water (56). Oligotrichs are mostly mixotrophic, which, thus, prefer upper water to perform photoautotrophic nutrition (Fig. S3D). In terms of feeding habit composition, our result showed that the proportion of algivore ciliate significantly decreased and that of detrics increased from upper to deep water. This obviously attributed to the distribution variation of each food source, i.e., the density of alga was lower while that of detritus was higher in deep than upper water. Overall, our results demonstrated that the significant variations of environmental factors formed a spatial niche separation between upper and deep water, which, thus, lead to the different communities of ciliates.

### Comparison of environmental and temp-spatial effects on community variation

To figure out the main determinants of ciliate distribution in neSCS, the contribution of temp-spatial dimensions and environmental factors in community structuring was compared here. The VPA revealed that the variation of entire community was more explained by environmental factors than temp-spatial factors. These results are consistent with the findings in the East China Sea’s euphotic zone that protists are more structured by environment selection relative to spatial effect (42). The relative importance of environment and spatial effect relies on variation ranges of each factor, and strong variation can significantly improve the effect on community assembly (57). In our study, the sampling sites covered the whole neSCS area which contains various patches impacted by different environmental processes. For example, the coastal area was characterized by high nutrition because of continental input, whereas the deep water has lower physical and biotic factors due to effects of thermocline (27). Therefore, the variation range of environmental factors is wide in this area. The strong environmental different represents high selective pressure, leading to the larger contribution of environmental determining in structuring communities. However, we noticed that the proportion of variation explained by our factors was low, and thus, the discussion on the determinant of community is limited in comparison of effects of environment and spatial factors. The mechanism of community assembling is pending further study based on process analyses.

Among the temp-spatial factors, the contribution of vertical dimension (i.e., depth) to the community distribution was higher than that of season and that of horizontal dimension was lowest. This was correspond to the result of community dissimilarity comparison among these three dimensions, i.e., the community dissimilarity between vertical groups was more significant than seasonal groups and that between horizontal groups was the lowest (Fig. S2). Considering the stronger variation of environment characters in water column than seasonal and horizontal directions (Fig. S1), it is not surprising that the influences of depth on ciliate community were stronger than those of season and horizontal distance.

In addition, the influences of physical and chemical factors and food resources to ciliate community were compared, which showed that physical factors had largest contribution to the community variation, followed by food, while chemical factors had smallest contribution. The strong impact of physical factor on microzooplankton community was also revealed in the boundary zone of SCS (58). Physical factors were the main indicator of water masses, so their impacts on microzooplankton were explained as the ecological effect of water masses (58). In neSCS, there commonly occurred various physical processes which were considered the initial driver of most environmental variation (59). Therefore, it is not surprising that physical factor plays more important roles in community structuring than other factors. The significant impact of chemical factors on ciliate community was generally found in coastal water, which attributes to the high proportion of mixtrophic species in the community that can actively respond to the high input of nitrogen and phosphorus from the continent (19). In neSCS, however, the proportion of mixtrophic species was low, and the community was dominated by hetretrophic species. The community mostly gains nutrition from food rather than chemical factors, which probably explain the higher contributions of food than chemical factors.

Considering the significant variation of subcommunities, their determinant was analyzed, respectively. VPA showed that the relative influences of temp-spatial dimensions and environmental factors on community were similar for summer and winter, which were both correspond with the results of the entire community, suggesting that the roles of these factors do not vary with the season change. In the three spatial areas, the season became the main driver for the variation of subcommunities among the temp-spatial dimensions, and its explanation power is higher in neritic water and upper oceanic water than the entire community. This suggests that the influences of season on communities enhanced with the decrease of spatial scale. Indeed, the seasonal variations of community were revealed in each area by our ANOSIM with a higher R value than the entire neSCS. Following the same reason of the seasonal variations of the entire community as mentioned above, the seasonal fluctuations of physical environment process may also be responsible for seasonal variations of subcommunities. Furthermore, these processes were region-specific. For example, the neritic water were controled by upwelling in summer but coastal current in winter, while upper oceanic water were effected by SCS current in summer but Kuroshio in winter (30). Therefore, the seasonal fluctuations of these processes were stronger in local area than entire neSCS and, thus, became the most important driver of subcommunities.

### Phytoplankton played important roles in the maintenance of community stability of ciliates

Previous studies have shown that modules comprise a subgrouping of species with similar environmental requirements in co-occurrence networks (60). The species clustering into distinct network modules has been used to infer physiochemical niches for various microbial groupings (61). In our network of ciliate community, M1 exhibited the highest degree among the module, suggesting the relationships among species in M1 were stronger. Notably, the abundances and niche breadths of the species were significantly higher in M1 than in other modules. This indicates that the abundant species generally have a wide niches range in the neSCS, which facilitates them to distribute widely and form the co-occurrence relationships and, thus, explain the high node degree. In addition, some modules displayed the different seasonal preferences. For example, M2, M4, and M5 were specific to summer, while M9 was specific to winter (Fig. S6). The community composition revealed that M2, M4, and M5 were dominated by Choreotrichs, but M9 was dominated by Litostomatea + Oligotrichs (Fig. S7), which well matched with the dominants of summer and winter subcommunties (Fig. 4). This indicates that the seasonal preferences of co-occurrences in the module might depend on their dominance in each season.

It has been known that phytoplankton were the direct determinant of ciliates in microbial food web, and the influence of environmental factors on ciliates was mediated indirectly by phytoplankton (4). In our study, we applied network analysis to explore the co-occurrence patterns of environment, phytoplankton, and ciliate communities in the neSCS. As expected, the ciliate displayed more relationships with phytoplankton than with environment. These confirmed that the phytoplankton played more important roles in the community construction of ciliates compared with environmental factors. Among the ciliates related with phytoplankton, the algivore (feeding on alges) account for the most proportion, suggesting the relations between ciliate and phytoplankton were mostly built based on predator-prey relationships. On the side of phytoplankton, diatom contributes a higher proportion to the relations than others. The extensive relationship between diatom and ciliate can be built in two aspects: diatom are the important prey sources of ciliates (62), and some diatom and ciliate can form symbiotic relationship by attaching each other (63). For the relations between ciliates and environmental factors, we found the high proportion associated with physical components, which further explained the high contributions of physical factors to the community distribution as revealed in VPA. In addition, the relations referring food all come from hetreotrophic ciliates, while mixotrophic species were only related to physical and chemical factors, which reflected their trophic preference.

### Conclusion

In the present study, we obtained a data set of ciliate communities with fine scale sampling covering continental shelf to the deep basin and revealed that spatial area, seasonality, and environmental variations defined the community pattern of ciliate in neSCS. The seasonal variations were not significant for alpha diversity but significant for beta diversity. For spatial dimension, alpha diversity and abundance decreased from neritic water to deep, and the community composition displayed significant variation between upper and deep water. Compared with temp-spatial factors, environmental factors take more responsibilities for the distribution of ciliate community. Among the environmental factors, physical factors showed the largest contribution to community variation. The ciliate displayed more relationships with phytoplankton than with environment factors in the network, indicating that phytoplankton played more important roles in the maintenance of community stability of ciliates compared with environmental factors. In the ciliate-environment co-occurrence relationships, physical factors contributed most. Our findings suggested that the physical processes play key roles in temp-spatial dynamics of ciliate community in open ocean.

## Data Availability

The ciliate community data are openly available in Science Data Bank at https://doi.org/10.57760/sciencedb.14038.

## SUPPLEMENTAL MATERIAL

Supplemental material spectrum.01247-24-s0001.docx Table S1; Fig. S1 to S7.

ASM does not own the copyrights to Supplemental Material that may be linked to, or accessed through, an article. The authors have granted ASM a non-exclusive, world-wide license to publish the Supplemental Material files. Please contact the corresponding author directly for reuse.

## References

[1] de Vargas C, Audic S, Henry N, Decelle J, Mahé F, Logares R, Lara E, Berney C, Le Bescot N, Probert I, et al.. 2015. Ocean plankton. Eukaryotic plankton diversity in the sunlit ocean. Science 348:1261605. doi:10.1126/science.1261605 25999516

[2] Gimmler A, Korn R, de Vargas C, Audic S, Stoeck T. 2016. The Tara Oceans voyage reveals global diversity and distribution patterns of marine planktonic ciliates. Sci Rep 6:33555. doi:10.1038/srep33555 27633177 PMC5025661

[3] Lynn D. 2008. The ciliated protozoa: characterization, classification, and guide to the literature. Third ed. Springer, New York.

[4] Azam F, Malfatti F. 2007. Microbial structuring of marine ecosystems. Nat Rev Microbiol 5:782–791. doi:10.1038/nrmicro1747 17853906

[5] Caron DA. 2017. Acknowledging and incorporating mixed nutrition into aquatic protistan ecology, finally. Environ Microbiol Rep 9:41–43. doi:10.1111/1758-2229.12514 28019711

[6] Campello-Nunes PH, Woelfl S, da Silva-Neto ID, da S. Paiva T, Fernández LD. 2022. Checklist, diversity and biogeography of ciliates (Ciliophora) from Chile. Eur J Protistol 84:125892. doi:10.1016/j.ejop.2022.125892 35436680

[7] Huang H, Yang J, Huang S, Gu B, Wang Y, Wang L, Jiao N, Xu D. 2021. Spatial distribution of planktonic ciliates in the western Pacific Ocean: along the transect from Shenzhen (China) to Pohnpei (Micronesia). Mar Life Sci Technol 3:103–115. doi:10.1007/s42995-020-00075-7 37073387 PMC10077192

[8] Santoferrara LF, Qureshi A, Sher A, Blanco-Bercial L. 2023. The photic-aphotic divide is a strong ecological and evolutionary force determining the distribution of ciliates (Alveolata, Ciliophora) in the ocean. J Eukaryot Microbiol 70:e12976. doi:10.1111/jeu.12976 37029732

[9] Sidi Ali R, Khames GEY, Alioua Z, Seridji R. 2023. Influence of environmental factors on biodiversity, abundance and the distribution pattern of dinoflagellates and ciliates during spring and summer in coastal waters of Algeria (southwestern Mediterranean Sea). J Mar Biol Ass 103. doi:10.1017/S0025315423000371

[10] Xu Y, Soininen J. 2019. Spatial patterns of functional diversity and composition in marine benthic ciliates along the coast of China. Mar Ecol Prog Ser 627:49–60. doi:10.3354/meps13086

[11] Liu W, McManus GB, Lin X, Huang H, Zhang W, Tan Y. 2021. Distribution patterns of Ciliate Diversity in the South China Sea. Front Microbiol 12:689688. doi:10.3389/fmicb.2021.689688 34539599 PMC8446678

[12] Xu Y, Chen X, Zhang J, Soininen J. 2022. Regional and local environment drive biogeographic patterns in intertidal microorganisms. J Biogeogr 49:1576–1585. doi:10.1111/jbi.14460

[13] Martiny JBH, Bohannan BJM, Brown JH, Colwell RK, Fuhrman JA, Green JL, Horner-Devine MC, Kane M, Krumins JA, Kuske CR, Morin PJ, Naeem S, Ovreås L, Reysenbach A-L, Smith VH, Staley JT. 2006. Microbial biogeography: putting microorganisms on the map. Nat Rev Microbiol 4:102–112. doi:10.1038/nrmicro1341 16415926

[14] Zhou J, Ning D. 2017. Stochastic community assembly: does it matter in microbial ecology? Microbiol Mol Biol Rev 81:1–32. doi:10.1128/MMBR.00002-17 PMC5706748 29021219

[15] Forster D, Behnke A, Stoeck T. 2012. Meta-analyses of environmental sequence data identify anoxia and salinity as parameters shaping ciliate communities. System Biodivers 10:277–288. doi:10.1080/14772000.2012.706239

[16] Wang C, Zhao Y, Du P, Ma X, Li S, Li H, Zhang W, Xiao T. 2022. Planktonic ciliate community structure and its distribution in the oxygen minimum zones in the Bay of Bengal (eastern Indian Ocean). J Sea Res 190:102311. doi:10.1016/j.seares.2022.102311

[17] Wang Y, Zhang W, Lin Y, Zheng L, Cao W, Yang J. 2013. Spatial pattern of the planktonic ciliate community and its relationship with the environment in spring in the northern Beibu Gulf, South China sea. Oceanol Hydrobiol Stud 42:470–479. doi:10.2478/s13545-013-0103-x

[18] Wang Y, Zhang W, Lin Y, Cao W, Zheng L, Yang J. 2014. Phosphorus, nitrogen and chlorophyll-a are significant factors controlling ciliate communities in summer in the Northern Beibu Gulf, South China sea. PLoS ONE 9:e101121. doi:10.1371/journal.pone.0101121 24987960 PMC4079230

[19] Wickham SA, Claessens M, Post AF. 2015. Ciliates, microbes and nutrients: interactions in the seasonally mixed Gulf of Aqaba. J Plankton Res 37:258–271. doi:10.1093/plankt/fbu103

[20] Li J, Chen F, Liu Z, Zhao X, Yang K, Lu W, Cui K. 2016. Bottom-up versus top-down effects on ciliate community composition in four eutrophic lakes (China). Eur J Protistol 53:20–30. doi:10.1016/j.ejop.2015.12.007 26773905

[21] Sun P, Wang Y, Laws E, Huang B. 2020. Water mass-driven spatial effects and environmental heterogeneity shape microeukaryote biogeography in A subtropical, hydrographically complex ocean system - A case study of ciliates. Sci Total Environ 706:135753. doi:10.1016/j.scitotenv.2019.135753 31836222

[22] Rakshit D, Biswas SN, Sarkar SK, Bhattacharya BD, Godhantaraman N, Satpathy KK. 2014. Seasonal variations in species composition, abundance, biomass and production rate of tintinnids (Ciliata: Protozoa) along the Hooghly (Ganges) River Estuary, India: a multivariate approach. Environ Monit Assess 186:3063–3078. doi:10.1007/s10661-013-3601-9 24402056

[23] Dolan JR, Montagnes DJS, Agatha S, Coats W, Stoecker DK. 2012. The biology and ecology of tintinnid ciliates: models for marine plankton. Wiley, Oxford.

[24] Grattepanche J-D, McManus GB, Katz LA. 2016. Patchiness of ciliate communities sampled at varying spatial scales along the New England shelf. PLoS One 11:e0167659. doi:10.1371/journal.pone.0167659 27936137 PMC5147948

[25] Yang J, Huang S, Fan W, Warren A, Jiao N, Xu D. 2020. Spatial distribution patterns of planktonic ciliate communities in the East China Sea: potential indicators of water masses. Mar Pollut Bull 156:111253. doi:10.1016/j.marpolbul.2020.111253 32510395

[26] Dai M, Meng F, Tang T, Kao SJ, Lin J, Chen J, Huang JC, Tian J, Gan J, Yang S. 2009. Excess total organic carbon in the intermediate water of the South China sea and its export to the North Pacific. Geochem Geophys Geosyst 10:1–17. doi:10.1029/2009GC002752

[27] Jilan S. 2004. Overview of the South China sea circulation and its influence on the coastal physical oceanography outside the pearl river Estuary. Cont Shelf Res 24:1745–1760. doi:10.1016/j.csr.2004.06.005

[28] Hu J, Kawamura H, Hong H, Qi Y. 2000. A review on the currents in the South China Sea: seasonal circulation, South China sea warm current and kuroshio intrusion. J Oceanogr 56:607–624. doi:10.1023/A:1011117531252

[29] Huang D, Zhang X, Jiang Z, Zhang J, Arbi I, Jiang X, Huang X, Zhang W. 2017. Seasonal fluctuations of ichthyoplankton assemblage in the northeastern South China Sea influenced by the Kuroshio intrusion. J Geophys Res Oceans 122:7253–7266. doi:10.1002/2017JC012906

[30] Ding X, Liu J, Zhang H, Ke Z, Li J, Liu W, Li K, Zhao C, Tan Y. 2022. Phytoplankton community patterns in the Northeastern South China Sea: implications of intensified kuroshio intrusion during the 2015/16 El Niño. JGR Oceans 127:e2021JC017998. doi:10.1029/2021JC017998

[31] Li J, Jiang X, Li G, Jing Z, Zhou L, Ke Z, Tan Y. 2017. Distribution of picoplankton in the northeastern South China Sea with special reference to the effects of the Kuroshio intrusion and the associated mesoscale eddies. Sci Total Environ 589:1–10. doi:10.1016/j.scitotenv.2017.02.208 28273592

[32] Kofoid CA, Campbell AS. 1929. A conspectus of the marine and fresh-water ciliata belonging to suborder Tintinnoinea, with descriptions of new species principally from the Agassiz expedition to the Eastern Tropical Pacific 1904-1905. Univ Calif Publ Zool 34:1–403.

[33] Hu X, Lin X, Song W. 2019. Ciliate atlas: species found in the South China sea. Science Press, Beijing.

[34] Song W, Warren A, Hu X. 2009. Free-living ciliates in the bohai and yellow seas, China. Science Press, Beijing.

[35] Wilbert N. 1975. Eine verbesserte technik der protargolimprägnation für ciliaten. Mikrokosmos 64:171–179.

[36] Fernandez-Leborans G, Fernandez-Fernandez D. 2002. Protist functional groups in a sublittoral estuarine epibenthic area. Estuaries 25:382–392. doi:10.1007/BF02695981

[37] Pratt JR, Cairns J. 1985. Functional groups in the protozoa: roles in differing ecosystems. J Protozool 32:415–423. doi:10.1111/j.1550-7408.1985.tb04037.x

[38] Foissner W, Berger H, Schaumburg J. 1999. Identification and ecology of limnetic plankton ciliates. Informationsberichte des Bayer, Landesamtes für Wasserwirtschaft.

[39] R Core Team. 2021. R: a language and environment for statistical computing. Vienna, Austria R Foundation

[40] Erdös P, Rényi A. 1960. On the evolution of random graphs. Publ Math Inst Hung Acad Sci 5:17–61.

[41] Zhu C, Liu W, Li X, Xu Y, El‐Serehy HA, Al‐Farraj SA, Ma H, Stoeck T, Yi Z. 2021. High salinity gradients and intermediate spatial scales shaped similar biogeographical and co‐occurrence patterns of microeukaryotes in a tropical freshwater‐saltwater ecosystem. Environ Microbiol 23:4778–4796. doi:10.1111/1462-2920.15668 34258839

[42] Wu W, Lu H-P, Sastri A, Yeh Y-C, Gong G-C, Chou W-C, Hsieh C-H. 2018. Contrasting the relative importance of species sorting and dispersal limitation in shaping marine bacterial versus protist communities. ISME J 12:485–494. doi:10.1038/ismej.2017.183 29125596 PMC5776463

[43] Bojanić N, Šolić M, Krstulović N, Šestanović S, Marasović I, Ninčević Ž. 2005. Temporal variability in abundance and biomass of ciliates and copepods in the eutrophicated part of Kaštela Bay (Middle Adriatic Sea). Helgol Mar Res 59:107–120. doi:10.1007/s10152-004-0199-x

[44] Santoferrara L, Alder V. 2009. Abundance trends and ecology of planktonic ciliates of the south-western Atlantic (35-63 S): a comparison between neritic and oceanic environments. J Plankton Res 31:837–851. doi:10.1093/plankt/fbp033

[45] Doherty M, Tamura M, Costas BA, Ritchie ME, McManus GB, Katz LA. 2010. Ciliate diversity and distribution across an environmental and depth gradient in long Island sound, USA. Environ Microbiol 12:886–898. doi:10.1111/j.1462-2920.2009.02133.x 20113332

[46] Sun P, Huang L, Xu D, Warren A, Huang B, Wang Y, Wang L, Xiao W, Kong J. 2019. Integrated space-time dataset reveals high diversity and distinct community structure of ciliates in mesopelagic waters of the Northern South China sea. Front Microbiol 10:2178. doi:10.3389/fmicb.2019.02178 31616397 PMC6768975

[47] Wang C, Li H, Zhao L, Zhao Y, Dong Y, Zhang W, Xiao T. 2019. Vertical distribution of planktonic ciliates in the oceanic and slope areas of the western Pacific Ocean. Deep Sea Res Part II: Top Stud Oceanogr 167:70–78. doi:10.1016/j.dsr2.2018.08.002

[48] Bachy C, Moreira D, Dolan JR, López-García P. 2014. Seasonal dynamics of free-living tintinnid ciliate communities revealed by environmental sequences from the North-West mediterranean sea. FEMS Microbiol Ecol 87:330–342. doi:10.1111/1574-6941.12224 24117776

[49] Countway PD, Vigil PD, Schnetzer A, Moorthi SD, Caron DA. 2010. Seasonal analysis of protistan community structure and diversity at the USC microbial observatory (san Pedro Channel, North Pacific Ocean). Limnol Oceanogr 55:2381–2396. doi:10.4319/lo.2010.55.6.2381

[50] Kim DY, Countway PD, Jones AC, Schnetzer A, Yamashita W, Tung C, Caron DA. 2014. Monthly to interannual variability of microbial eukaryote assemblages at four depths in the eastern North Pacific. ISME J 8:515–530. doi:10.1038/ismej.2013.173 24173457 PMC3930315

[51] Zhang W, Chen X, Li H, Wang C, Liang C, Zhang S. 2016. Marine planktonic ciliates grazing: a review. Oceanol Limnol Sin 47:276–289. doi:10.11693/hyhz20150600160

[52] Lee Chen Y, Chen H-Y, Tuo S, Ohki K. 2008. Seasonal dynamics of new production from Trichodesmium N2 fixation and nitrate uptake in the upstream Kuroshio and South China Sea basin. Limnol Oceanogr 53:1705–1721. doi:10.4319/lo.2008.53.5.1705

[53] Santoferrara LF, Grattepanche J-D, Katz LA, McManus GB. 2016. Patterns and processes in microbial biogeography: do molecules and morphologies give the same answers? ISME J 10:1779–1790. doi:10.1038/ismej.2015.224 26849313 PMC4918432

[54] Zhao F, Filker S, Xu K, Huang P, Zheng S. 2017. Patterns and drivers of vertical distribution of the ciliate community from the surface to the abyssopelagic zone in the Western Pacific Ocean. Front Microbiol 8:2559. doi:10.3389/fmicb.2017.02559 29312240 PMC5742212

[55] Gómez F. 2007. Trends on the distribution of ciliates in the open Pacific Ocean. Acta Oecol 32:188–202. doi:10.1016/j.actao.2007.04.002

[56] Agatha S, Simon P. 2012. On the nature of tintinnid loricae (Ciliophora: Spirotricha: Tintinnina): a histochemical, enzymatic, edx, and high-resolution TEM Study. Acta Protozool 51:1–19. doi:10.4467/16890027AP.12.001.0384 22988335 PMC3442249

[57] Logares R, Tesson SVM, Canbäck B, Pontarp M, Hedlund K, Rengefors K. 2018. Contrasting prevalence of selection and drift in the community structuring of bacteria and microbial eukaryotes. Environ Microbiol 20:2231–2240. doi:10.1111/1462-2920.14265 29727053

[58] Sun P, Zhang S, Wang Y, Huang B. 2021. Biogeographic role of the kuroshio current intrusion in the microzooplankton community in the boundary zone of the Northern South China Sea. Microorganisms 9:1104. doi:10.3390/microorganisms9051104 34065542 PMC8161332

[59] Nan F, Xue H, Yu F. 2015. Kuroshio intrusion into the South China Sea: a review. Prog Oceanogr 137:314–333. doi:10.1016/j.pocean.2014.05.012

[60] Borthagaray AI, Arim M, Marquet PA. 2012. Connecting landscape structure and patterns in body size distributions. Oikos 121:697–710. doi:10.1111/j.1600-0706.2011.19548.x

[61] Steele JA, Countway PD, Xia L, Vigil PD, Beman JM, Kim DY, Chow C-E, Sachdeva R, Jones AC, Schwalbach MS, Rose JM, Hewson I, Patel A, Sun F, Caron DA, Fuhrman JA. 2011. Marine bacterial, archaeal and protistan association networks reveal ecological linkages. ISME J 5:1414–1425. doi:10.1038/ismej.2011.24 21430787 PMC3160682

[62] Montagnes DJS, Allen J, Brown L, Bulit C, Davidson R, Fielding S, Heath M, Holliday NP, Rasmussen J, Sanders R, Waniek JJ, Wilson D. 2010. Role of ciliates and other microzooplankton in the Irminger Sea (NW Atlantic Ocean). Mar Ecol Prog Ser 411:101–115. doi:10.3354/meps08646

[63] Gómez F. 2020. Symbioses of ciliates (Ciliophora) and diatoms (Bacillariophyceae): taxonomy and host–symbiont interactions. Oceans 1:133–155. doi:10.3390/oceans1030010

