# Supplementary material for: Effects of temporal, spatial, and environmental factors on ciliates community in northeastern South China Sea, with notes on co-occurrence patterns of environment, phytoplankton, and ciliate
Source: Microbiol Spectr. 2024 Nov 29;13(1):e01247-24. doi: 10.1128/spectrum.01247-24 (PMC11705822; doi:10.1128/spectrum.01247-24)
Supplement: Supplemental material — Table S1; Fig. S1 to S7. [file spectrum.01247-24-s0001.docx]

Supporting Information

**Table. S1.** Comparison of topological properties of node-level co-occurrence networks of ciliate communities and ciliate-phytoplankton-environment integrated data with their associated random networks.

|  | Nodes | Edges | avgCC | avgPL | Modularity | r* |
| --- | --- | --- | --- | --- | --- | --- |
| Ciliate network | 167 | 451 | 0.3367 | 3.9141 | 0.6041 | 32.8 |
| Random network | 167 | 451 | 0.0325 | 3.1996 | 0.0325 |  |
| Integrated network | 199 | 686 | 0.3535 | 3.6523 | 0.4961 | 8.21 |
| Random network | 199 | 686 | 0.0347 | 2.9734 | 0.3421 |  |

avgCC, average clustering coefficient; avgPL, average path length; r, Small-word coefficient, r>1 indicates “small-world” properties, that is, high interconnectivity and high efficiency.

**Figure S1-S7.**


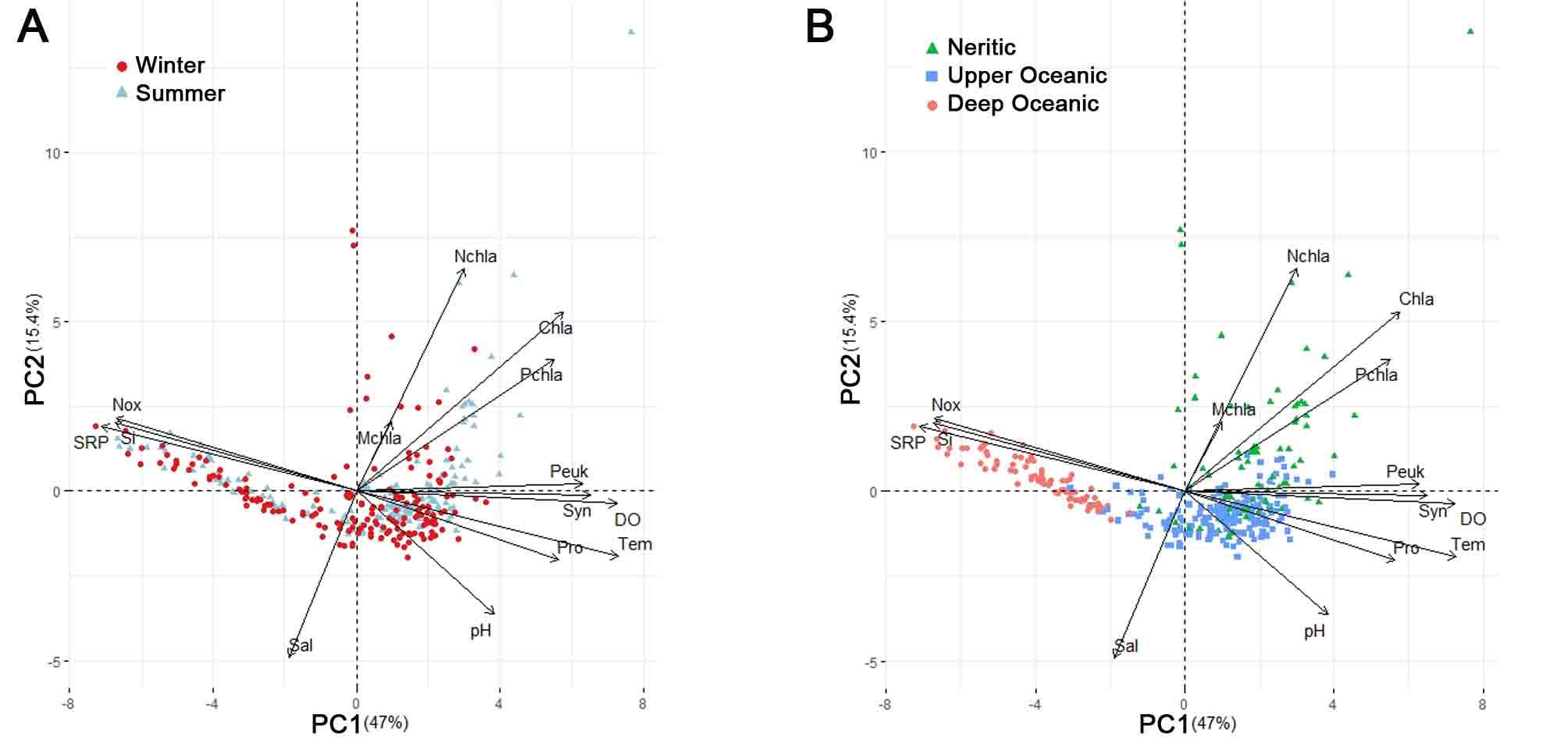


Fig. S1. Principal component analysis (PCA) showing the resemblance of environmental factors, samples coded by color in the two seasons (A) and three spatial zones (B). Tem: temperature; Sal: salinity; DO: dissolved oxygen; Nox: inorganic nitrogen; SRP: soluble reactive phosphorus; Si: active silicon; Chla: total chlorophyll a; MChla: microplankton chlorophyll a (>20 μm); NChla: nanoplankton chlorophyll a (3-20 μm); PChla: picoplankton chlorophyll a (<3 μm); Syn: picoplanktons Synechococcus; Peuk: Picoeukaryotes; and Pro: Prochlorococcus.


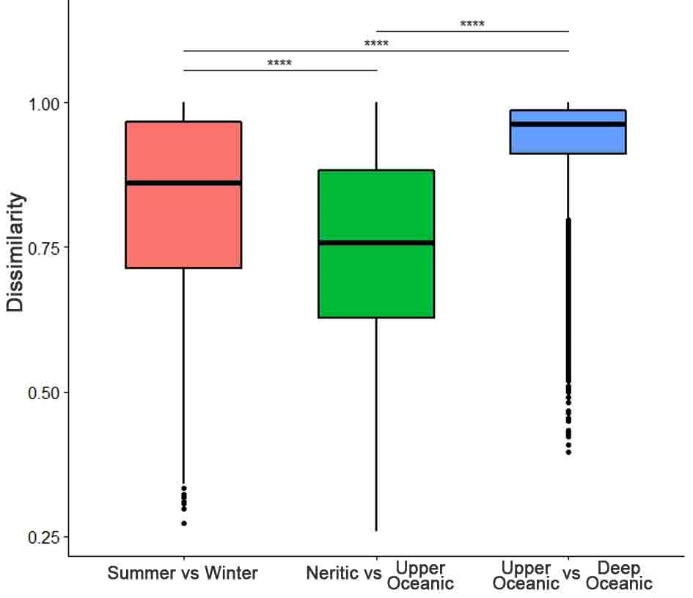


Fig. S2. The community dissimilarity among season and spatial groupings according to pairwise Bray-Curtis dissimilarity.


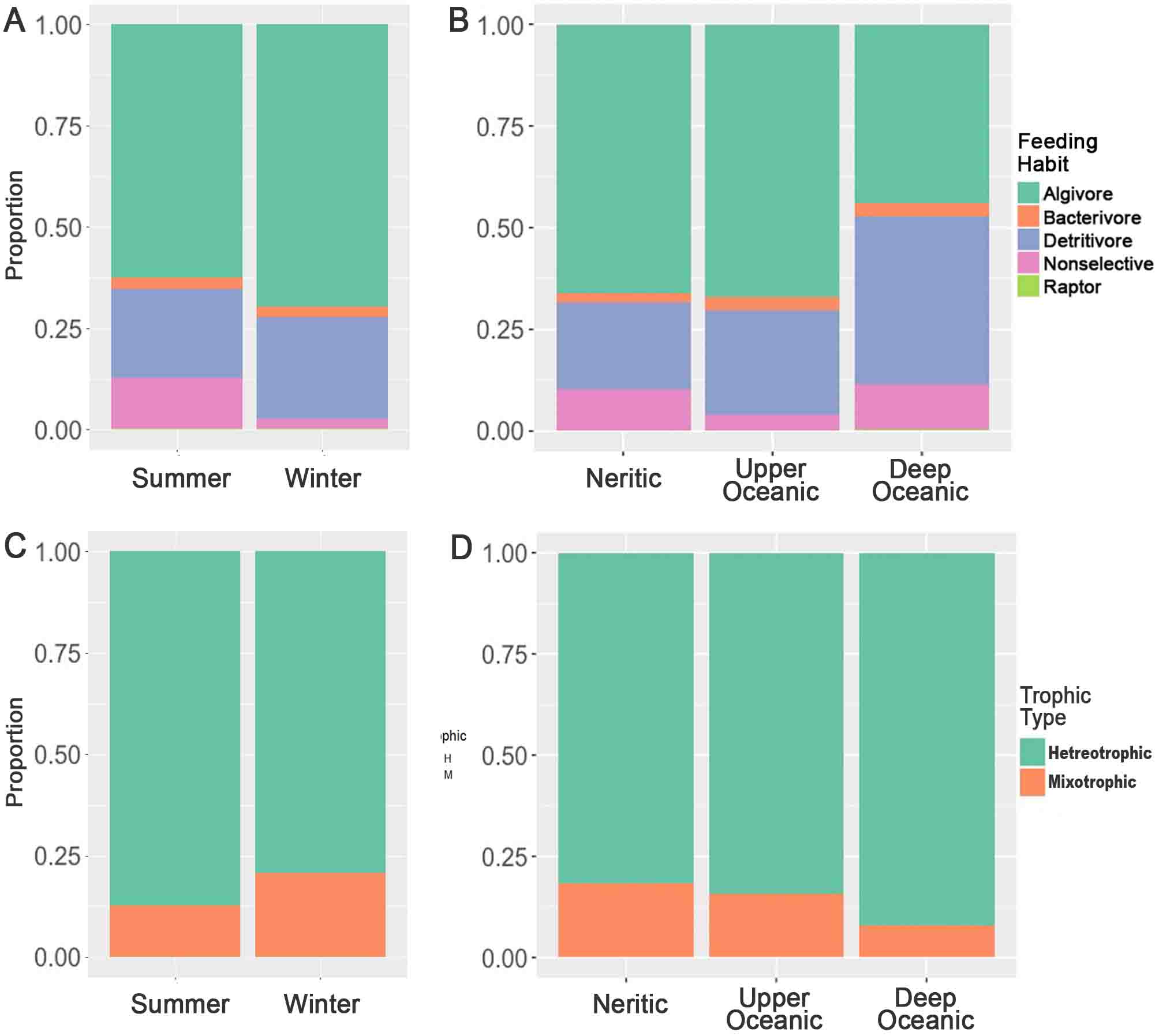


Fig. S3. Compositions of feeding habit grouping of ciliates in two seasons (A) and in three spatial zones (B). Compositions of trophic type grouping of ciliates in two seasons (C) and in three spatial zones (D).


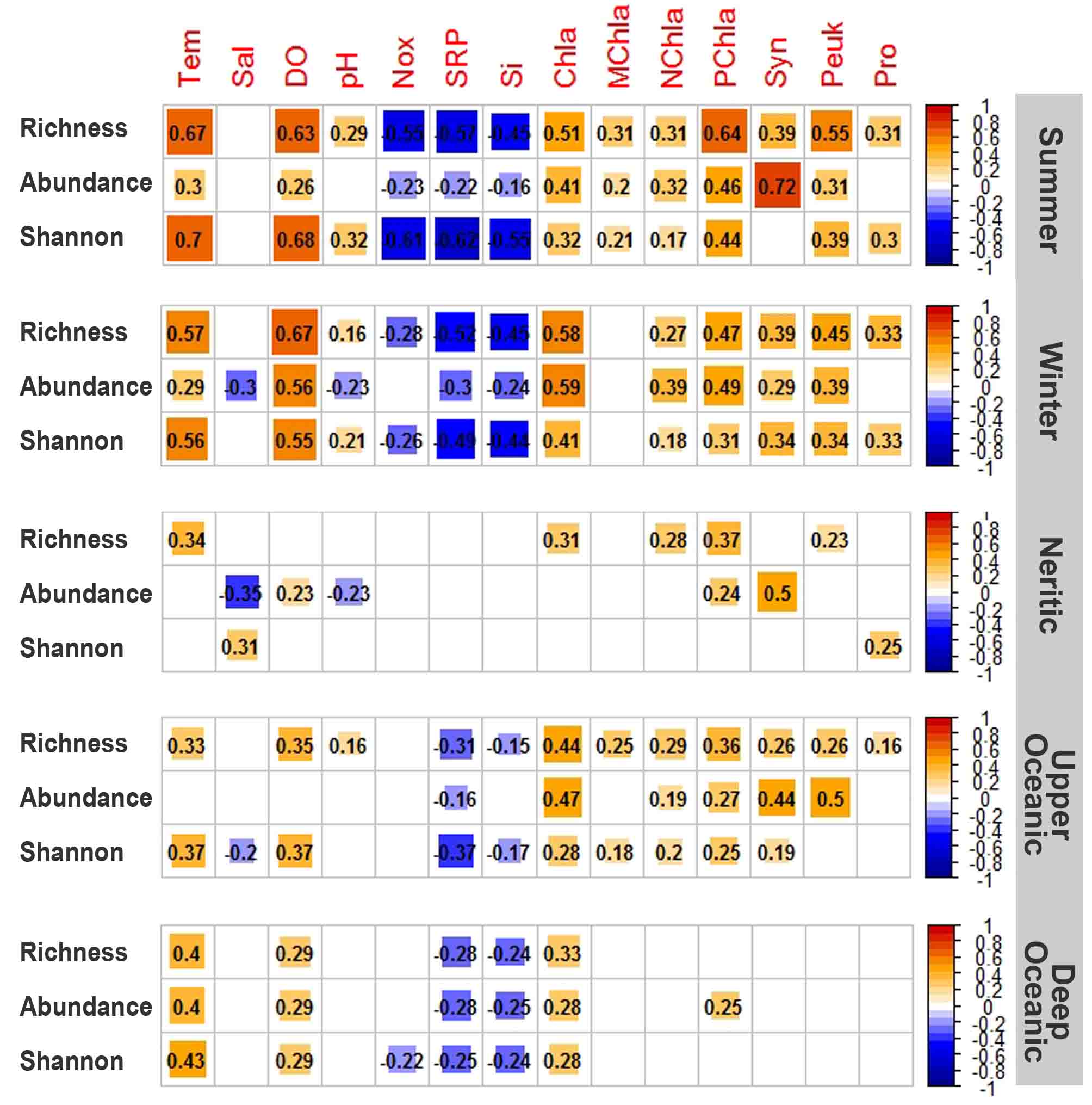


Fig. S4. Correlation analyses between environmental variables and ciliate species richness, abundance and shannon in each temp-spatial subcommunity. The number and color gradient representing Pearson’s correlation coefficients. Tem: temperature; Sal: salinity; DO: dissolved oxygen; NOx: inorganic nitrogen; SRP: soluble reactive phosphorus; Si: active silicon; Chla: total chlorophyll a; Mchla: microplankton chlorophyll a (>20 μm); NChla: nanoplankton chlorophyll a (3-20 μm); PChla: picoplankton chlorophyll a (<3 μm); Syn: picoplanktons Synechococcus; Peuk: Picoeukaryotes; and Pro: Prochlorococcus.


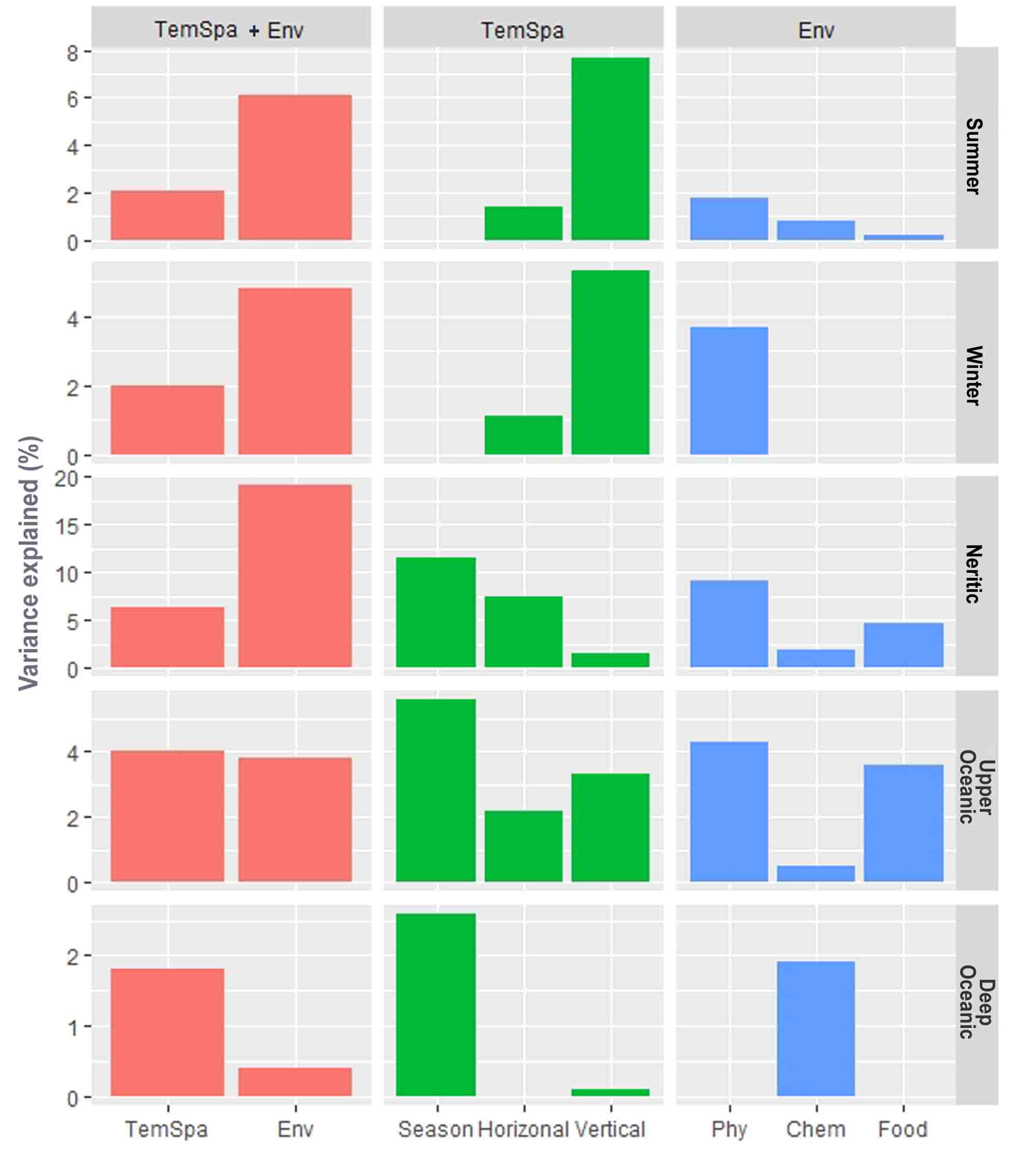


Fig. S5. Comparisons of the explanatory power of various drivers for each temp-spatial subcommunity revealed by variation partitioning analysis. TemSpa: temp-spatial factors referring to season, depth (vertical) and PCNM (horizontal); Env: environmental factors referring to physical (Phy: Sal, Tem, pH, DO), chemical (Chem: Nox, SRP, Si) and food (MChla, PChla, NChla, Syn, Peuk, and Pro) variables. Forward selection procedures were used to select the best subset of variables explaining community variation, respectively.


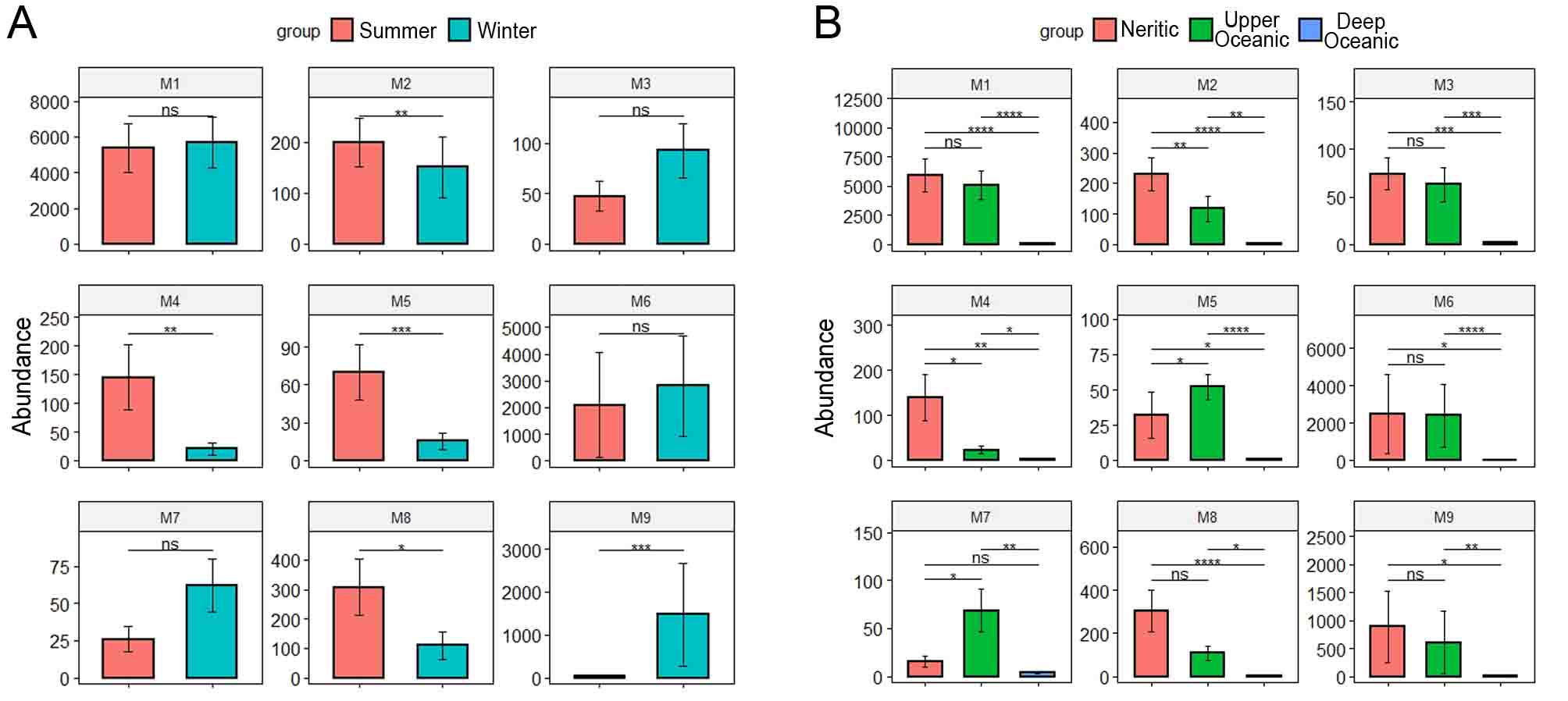


Fig. S6. Comparisons of average abundance of nodes among the season (A) and spatial (B) groupings for each module of the co-occurrence network.


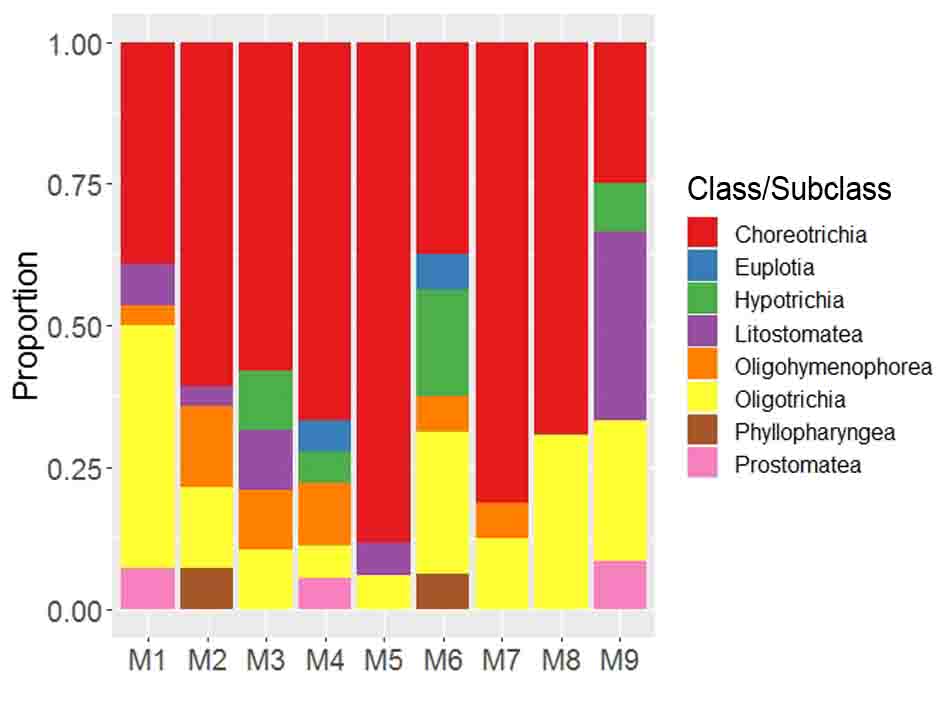


Fig. S7. Taxonomic composition of ciliates in each module of the co-occurrence network at class/subclass level
